# Disorders of the Basal Ganglia and Cerebellum: A Comprehensive Review of Speech Sensorimotor Symptoms and Responses to Feedback Perturbations

**DOI:** 10.1162/NOL.a.291

**Published:** 2026-09-14

**Authors:** Hantao Wang, Ludo Max

**Affiliations:** Department of Speech and Hearing Sciences, University of Washington, Seattle, WA, USA

**Keywords:** basal ganglia, cerebellum, essential tremor, sensorimotor integration, speech motor control, Parkinson’s disease

## Abstract

Situated within critical cortical-subcortical sensorimotor circuits, the basal ganglia and cerebellum are thought to play important roles in both feedforward and feedback mechanisms of speech sensorimotor control. Motor speech impairments and speech-related sensory deficits are indeed common in disorders involving these two structures such as Parkinson’s disease, dystonia, and cerebellar degeneration. Recently, several studies have also investigated the impact of these disorders on the feedforward and feedback components of speech sensorimotor control by examining immediate compensation to unexpected auditory perturbations (auditory-motor compensation) and across-trials adaptation to predictable auditory perturbations (auditory-motor adaptation). Additional studies have investigated how the speech of patients with these disorders is affected by pharmacological interventions and deep brain stimulation of neural substrates related to the basal ganglia or cerebellum. This review summarizes and integrates the growing body of literature based on these three lines of inquiry regarding speech sensorimotor control in basal ganglia and cerebellar disorders: (a) motor and sensory impairments, (b) auditory-motor compensation and adaptation, and (c) effects of pharmacological and deep brain stimulation interventions on symptoms and on auditory-motor compensation/adaptation. The review highlights areas of consistency and inconsistency in the obtained results, demonstrates an apparent dissociation between impact on the phonatory versus articulatory subsystems, and identifies several critical gaps in our knowledge to date. Taken together, these insights call for further studies on speech sensorimotor control in patients with basal ganglia and cerebellar disorders, in particular studies using more rigorous experimental controls as well as innovative neuroimaging and neurostimulation techniques.

## INTRODUCTION

The basal ganglia and the cerebellum have long been recognized as critical subcortical structures for producing speech. Various forms of *motor speech impairment* have been observed in individuals with different disorders of the basal ganglia and the cerebellum (Duffy, 2013). Several of these disorders have also been known to lead to *deficits in speech-related sensory processes*, such as auditory processing and orofacial somatosensation (Clark et al., 2014; Hammer & Barlow, 2010). Furthermore, contemporary computational models of speech production assign to the basal ganglia and cerebellum critical roles in the implementation of feedforward and feedback control mechanisms (Parrell & Houde, 2019; Tourville & Guenther, 2011).

Feedforward control refers to the execution of preplanned motor commands that are predicted to satisfy the movement goals by internal models or a control policy (Haith & Krakauer, 2013; Miall & Wolpert, 1996; Shadmehr & Mussa-Ivaldi, 1994). The feedforward control mechanism can update future motor commands based on previously experienced sensory information. Feedback control refers to the modification of ongoing motor commands based on sensory error, i.e., a discrepancy between incoming sensory feedback and a sensory prediction generated based on the issued motor commands (Miall & Wolpert, 1996). In speech motor control, the sensory errors are usually thought to be auditory and/or somatosensory (Parrell & Houde, 2019; Tourville & Guenther, 2011). Because the basal ganglia and the cerebellum are in closed-loop circuits with cortical motor areas (the cortico-basal ganglia-thalamo-cortical loop and the cortico-cerebello-thalamo-cortical loop, respectively), they are well-positioned to implement such control mechanisms. In computational models such as the Directions Into Velocities of Articulators model, the basal ganglia play a key role in feedforward control (by selecting and initiating the appropriate motor commands through the integration of contextual cues from motor and nonmotor cortices), whereas the cerebellum is vital in both feedback control (monitoring sensory error) and feedforward control (updating internal models based on sensory error) (Tourville & Guenther, 2011).

These control mechanisms are often investigated in studies that implement sensory perturbations during speech production, most commonly perturbations that generate error in the auditory domain. On the one hand, to study *feedback* control, speakers are asked to prolong their speech while auditory perturbations (e.g., temporal or spectral shifts) are introduced *unexpectedly* in the auditory feedback. Most speakers exhibit *auditory-motor compensation* to counter the perturbation. Such compensatory behavior reflects online corrections made by the control system based on the perceived auditory error (Burnett et al., 1998; Purcell & Munhall, 2006). On the other hand, to study *feedforward* control, an auditory perturbation is implemented consistently—and, thus, *predictably*—in every trial of a perturbation phase during the experiment. Most speakers gradually (over several trials) change their speech movements to counter the perturbation, an adaptive behavior known as *auditory-motor adaptation* (Houde & Jordan, 1998; Villacorta et al., 2007)^1^. Such adaptive behavior is thought to reflect updates to internal models of sensorimotor control (Hadjiosif et al., 2021; Haith & Krakauer, 2013). In theories of sensorimotor control, internal models refer to internal mappings between motor commands and sensory consequences; specifically, the inverse internal models calculate from the desired sensory consequences the central commands necessary to achieve those consequences, whereas the forward internal models calculate the predicted sensory consequences of a planned motor command (Kawato, 1999; Miall & Wolpert, 1996; Wolpert et al., 1995). In this framework, sensorimotor adaptation is usually thought to be achieved through recalibration of the forward model based on the persistent errors between the predicted and the actual sensory consequences of the motor commands (Haith & Krakauer, 2013), although it has recently been suggested that direct updates to the inverse model may also play a role in adaptation (Hadjiosif et al., 2021). Control of the different speech subsystems can be studied by perturbing different acoustic parameters in the auditory feedback, most commonly the fundamental frequency (*f*_*o*_) for studying the phonatory subsystem^2^ and the first two formant frequencies (F1, F2) for studying the articulatory subsystem^3^.

Given the importance of the basal ganglia and the cerebellum for speech sensorimotor control, a number of studies have begun to identify atypical auditory-motor compensation and adaptation among individuals with different types of basal ganglia and cerebellar disorders. Furthermore, some of these differences have been interpreted as potentially underlying the presence of dysarthria in these clinical populations (Sapir, 2014). In a subset of these disorders, studies are also examining whether feedforward and feedback control are differentially affected by deep brain stimulation (DBS) of neural structures in the cortical-subcortical circuits involving the basal ganglia and the cerebellum. In this review, we aim to provide a state-of-the-knowledge overview of this growing literature regarding auditory-motor compensatory and adaptive speech behaviors in patients with basal ganglia and cerebellar disorders, and to discuss the findings in light of the speech-related motor and sensory impairments associated with these disorders.

Specifically, we focus here on three disorders or groups of disorders: Parkinson’s disease, dystonia, and cerebellar degeneration. They were selected based on two criteria: (1) their pathophysiology primarily involves the basal ganglia or cerebellum; and (2) studies have been conducted to examine either auditory-motor compensation or auditory-motor adaptation, or both, in affected individuals. For each (group of) disorder(s), we first present an overview of the characteristic phonatory and articulatory motor speech symptoms and related sensory impairments. Next, we present the results from a systematic search for studies on auditory-motor compensation and adaptation for each condition, and the studies’ findings are summarized and discussed within the framework of feedforward and feedback control mechanisms. Throughout each section, the effects of pharmacological agents or DBS on the symptoms and auditory-motor compensation and adaptation are also addressed when such information is available.

## PARKINSON’S DISEASE

### Motor Speech Symptoms in PD

Parkinson’s disease (PD) is a movement disorder characterized by bradykinesia, rigidity, and rest tremor (Postuma et al., 2015). Its cause is unknown in most cases, but PD is primarily associated with significant loss of the dopaminergic neurons of the substantia nigra that projects to the striatum in the basal ganglia circuit (Hornykiewicz, 2002). Speech impairment is common in individuals with PD (IWPD), with up to 90% of IWPD eventually developing speech symptoms (Ho et al., 1998; Logemann et al., 1978). PD is considered the prototypic disease of hypokinetic dysarthria, a motor speech disorder characterized by overall reduced movement amplitude across all subsystems of speech production (Duffy, 2013).

Perceptually, the most widely reported features of hypokinetic dysarthria are reduced overall loudness, monopitch and monoloudness, and breathy and/or rough voice quality (Darley et al., 1969; Duffy, 2013), which have been confirmed by a large number of acoustic studies. Compared with age-matched controls, connected speech from IWPD shows a lower overall mean intensity (Holmes et al., 2000; Ma et al., 2015; Yang et al., 2020), reduced range and variability of fundamental frequency (*f*_*o*_) (Gamboa et al., 1997; Harel et al., 2004; Holmes et al., 2000; Jiménez-Jiménez et al., 1997; Ma et al., 2015; Midi et al., 2008; Skodda et al., 2011; Skodda & Schlegel, 2008; Yang et al., 2020), and reduced range and variability of intensity (Gamboa et al., 1997; Holmes et al., 2000; Jiménez-Jiménez et al., 1997; Ma et al., 2015; Yang et al., 2020). Regarding voice quality, some studies have reported a decreased harmonic to noise ratio (Gamboa et al., 1997; Jiménez-Jiménez et al., 1997; Rusz et al., 2011), increased jitter (Gamboa et al., 1997; Jiménez-Jiménez et al., 1997; Rahn et al., 2007; Rusz et al., 2011; Yang et al., 2020), and increased shimmer (Jiménez-Jiménez et al., 1997; Rusz et al., 2011). This is also confirmed by the high prevalence of laryngeal structural and movement differences (Hanson et al., 1984; E. Lin et al., 1999; Midi et al., 2008; Perez et al., 1996; Yücetürk et al., 2002). Atypical laryngeal electromyographic activity has also been reported in IWPD (Baker et al., 1998; Gallena et al., 2001; Luschei et al., 1999; Zarzur et al., 2007, 2014).

The most prominent articulatory characteristic of hypokinetic dysarthria is imprecise consonant articulation (Darley et al., 1969; Duffy, 2013). This has been described as a process of “spirantization,” in which consonants are produced with less constriction between the articulators (Logemann & Fisher, 1981). Acoustically, spirantization is inferred from the presence of phonation during the closure period of stop consonants (Ackermann & Ziegler, 1991; Karlsson & Hartelius, 2019; Skodda et al., 2013). Furthermore, acoustic studies on hypokinetic vowels have revealed reduced formant transitions (Forrest et al., 1989; Rosen et al., 2006), decreased formant slope (Kim et al., 2009; Rosen et al., 2006), and a reduced vowel space based on measured formant frequencies (Rusz et al., 2013; Tjaden & Wilding, 2004; Whitfield & Goberman, 2014; Whitfield & Mehta, 2019). These features reflect “articulatory undershoot,” i.e., the articulators are not fully reaching their target positions due to a limited range of motion or reduced speed (Logemann & Fisher, 1981). This interpretation is supported by kinematic studies that showed reduced amplitude and/or speed of lip and jaw movements in IWPD (Bandini et al., 2016; Darling & Huber, 2011; Forrest & Weismer, 1995; Kearney et al., 2017; Kuruvilla-Dugdale et al., 2020; Mefferd & Dietrich, 2019). Some electromyographic studies also reported patterns of rigidity in certain articulatory muscles (Hirose, 1986; Hunker et al., 1982; Leanderson et al., 1972).

#### Effects of dopaminergic medications on motor speech symptoms in PD

Dopaminergic medications, most commonly levodopa, a precursor to dopamine used as a dopamine replacement agent, are widely used to treat the motor symptoms of PD with great effectiveness (Gandhi & Saadabadi, 2023; Pringsheim et al., 2021). However, their influence on the speech motor symptoms is mixed. One review of more than thirty studies on voice quality in PD reported generally positive effects of levodopa on perceptual judgments, but with no consistent improvement in acoustic measures (Lechien et al., 2018). Levodopa was reported to improve consonant articulation in a longitudinal study (Rusz et al., 2016), and to increase extent and velocity of articulatory movements in some individuals (Thies et al., 2021). However, a large number of studies have only found minor or no effects of such medication on speech outcomes in PD (Cushnie-Sparrow et al., 2018; Tykalova et al., 2022; Vandana et al., 2021), and some even reported adverse effects associated with the long-term use of levodopa such as increased speech disfluency (Tykalová et al., 2015).

#### Effects of DBS on motor speech symptoms in PD

Over the past several decades, deep brain stimulation of the subthalamic nucleus (STN-DBS) and globus pallidus pars interna (GPi-DBS) has been increasingly used to improve motor symptoms in IWPD who do not, or who do no longer, respond to medication (Perestelo-Pérez et al., 2014). However, similar to the situation for dopaminergic medication, the effect of DBS on speech motor symptoms in PD remains inconclusive. Aldridge et al. (2016) completed a systematic review on speech outcomes of STN-DBS. Out of the 47 studies included, 25 reported speech improvements after DBS treatment (post-STN-DBS vs. pre-STN-DBS) or with DBS turned on (STN-DBS-ON vs. STN-DBS-OFF), such as increased vocal intensity and increased *f*_*o*_ range. On the other hand, 27 studies reported a deterioration of speech measures such as decreased speech intelligibility, decreased articulatory accuracy, and reduced articulation range. Aldridge et al. (2016) identified the lack of consistent inclusion criteria (e.g., in- or exclusion of patients on dopaminergic medications), the lack of unified speech outcome measures, and the lack of longitudinal study approaches as major factors contributing to the divergence in results. Overall, global measurements such as speech intelligibility and self-perception of speech quality tend to worsen post-STN-DBS or with STN-DBS-ON, whereas the effects on specific acoustic or kinematic measures vary widely across studies. A more recent systematic review on speech, voice and language outcomes following DBS in both PD and essential tremor patients also reported heterogeneous pattern of outcomes across DBS studies (Tabari et al., 2024). That review paper reported that bilateral and low-frequency DBS appeared to demonstrate a relative improvement for phonation and articulation, but overall long-term DBS exacerbated phonation and articulation deficits (Tabari et al., 2024). No consistent differences in speech outcomes between STN-DBS and GPi-DBS have been reported in the literature (Tabari et al., 2024; van Brenk et al., 2024).

A growing number of studies have begun to examine whether and how speech outcomes may be related to, or can be optimized by, specific DBS parameters, such as location of the electrode contacts, stimulation frequency, amplitude, pulse width, etc. (Abeyesekera et al., 2019; Fabbri et al., 2019; Grover et al., 2019; Knowles et al., 2018; Phokaewvarangkul et al., 2019). A particularly informative study was reported by Fenoy et al. (2017) who found that speech outcomes of STN-DBS were modulated by how much the dentatorubrothalamic tract was affected by the stimulation. The dentatorubrothalamic tract is a fiber tract that projects from the cerebellar deep nuclei to the contralateral red nucleus and the ventral intermediate (Vim) nucleus of the thalamus. The majority of their participants with prominent dentatorubrothalamic tract involvement in the stimulation showed deterioration in overall speech outcomes with STN-DBS-ON, whereas speech remained unchanged or improved among patients with minimal dentatorubrothalamic tract involvement (Fenoy et al., 2017). Interestingly, as reviewed below, DBS at the Vim nucleus (Vim-DBS) is a common treatment for essential tremor and is known to cause stimulation-induced dysarthria in many patients (Alomar et al., 2017). Thus, the dentatorubrothalamic tract may play a critical role in determining speech outcomes of DBS across different clinical populations.

#### Effects of altered auditory feedback on motor speech symptoms in PD

Altered auditory feedback, including feedback delays, spectral manipulations (frequency shifts), amplification, and masking noise, have been studied as potential strategies to alleviate motor speech symptoms in PD. This approach is partly inspired by the fluency-enhancing effect of altered auditory feedback in stuttering (Brendel et al., 2004). Broadly speaking, auditory-motor perturbation studies can be considered specific types of altered auditory feedback tasks, and feedback and feedforward control mechanisms have been hypothesized to explain the fluency-enhancing effects of altered auditory feedback in stuttering (Bradshaw et al., 2021). However, there are also important methodological differences between studies that investigate the potential clinical effects of altered auditory feedback and studies that investigate auditory-motor compensation and adaptation. Studies that investigate the potential clinical effects of altered auditory feedback typically apply large feedback perturbations, such as delays of 150 ms or all-frequency shifts of 600 cents (Brendel et al., 2004; Kaipa et al., 2024; Lowit et al., 2010) whereas studies that investigate auditory-motor compensation and adaptation typically apply much smaller perturbations, such as *f*_*o*_ or formant shifts of 100 or 200 cents. Auditory feedback perturbations of extremely large magnitude tend to cause auditory-motor compensation and adaptation responses to plateau or even decrease (Burnett et al., 1998; Liu et al., 2012; MacDonald et al., 2010). This may explain why clinical studies on altered auditory feedback typically examine only symptom reduction and not adaptation or compensation.

Positive effects of altered auditory feedback (most commonly with feedback delay and frequency shift) have included increased loudness, increased pitch variation, and decreased speech rate, whereas the effects of altered auditory feedback on intelligibility and naturalness ratings have been mixed (Brendel et al., 2004; Kaipa et al., 2024; Lowit et al., 2010). Improvement of vocal quality under masking noise and deterioration of vocal quality under amplification and delay were reported in one study (Coutinho et al., 2009). Overall, there is large inter-individual variability in how the speech of IWPD changes with altered auditory feedback and studies usually reported both responders and non-responders in their samples (Brendel et al., 2004; Lowit et al., 2010).

### Speech-Related Sensory Impairments in PD

Basal ganglia disorders such as PD and dystonia are known to also cause deficits specifically in sensory processing. Many studies have reported that patients with these disorders show increased thresholds for somatosensory temporal discrimination and reduced somatosensory evoked potentials in response to stimulation of the extremities (Lee et al., 2018; Rossini et al., 1998; Tinazzi et al., 2009), which may be caused by atypical sensory gating (Kaji et al., 2005). Only a few studies have investigated orofacial somatosensory processing in PD. IWPD exhibited poor performance in orofacial proprioceptive and tactile tests (Schneider et al., 1986), increased vocal fold somatosensory thresholds (Hammer & Barlow, 2010), and reduced tongue tip tactile acuity (Y.-W. Chen & Watson, 2017). Caligiuri and Abbs (1987) also found a heightened perioral reflex to mechanical displacements of perioral tissue. In some of these studies, specific laryngeal and lingual somatosensory deficits correlated with the severity of phonatory or articulatory impairments, illustrating the close relationship between speech-related sensory processing and motor symptoms (Y.-W. Chen & Watson, 2017; Hammer & Barlow, 2010).

IWPD have also been found to have auditory deficits. Given the distinct low vocal intensity in hypokinetic dysarthria, several studies have focused on loudness perception among IWPD. Clark et al. (2014) found that IWPD used a more restricted range in their loudness estimations, and Troche et al. (2012) and Richardson and Sussman (2019) reported poor loudness discrimination for pure tones and vowels. However, Dromey and Adams (2000) reported no differences between IWPD and controls in rating the loudness of warbled pure tones. Besides differences in loudness perception, IWPD have also been reported to show poor auditory acuity in tasks assessing pure-tone discrimination and sibilant contrast discrimination (Y.-W. Chen & Watson, 2017; Troche et al., 2012). The relation between such reduced auditory performance and the observed speech sensorimotor problems remains unclear given that no correlation has been found between auditory performance measures and the severity of hypokinetic dysarthria (Y.-W. Chen & Watson, 2017; Richardson & Sussman, 2019).

In addition to these psychophical testing differences, IWPD have been reported to show atypical activity in electroencephalographic (EEG) recordings of the brain response to auditory stimulation. For example, at the brainstem level, there have been reports of longer latencies in auditory brainstem responses (Shalash et al., 2017) and larger amplitudes of the frequency-following response to repetitions of speech syllables (Mollaei et al., 2022). At the cortical level, Lukhanina et al. (2009, 2010) found that, in a paired-click auditory-stimulation paradigm, compared to controls, IWPD’s N1-P2 response to the second click stimulus was similar to the response to the first click, whereas controls showed significantly attenuated N1-P2 response to the second click than to the first click. These results suggest that PD is associated with auditory disinhibition. Similarly, De Keyser et al. (2021) reported that, compared to controls, IWPD showed greater increases in the amplitude of the P2 component evoked by auditory stimuli with increasing intensity. Overall, frequency following response and cortical auditory-evoked potential (AEP) components appear to be either larger in amplitude or less inhibited in IWPD than in healthy controls.

Besides the above findings regarding the auditory processing of external stimuli, there exists a body of literature on the processing of self-generated sounds in IWPD. Clinicians usually observe that many IWPD are unaware of the low intensity level of their own speech, and that IWPD can increase their speech intensity to a typical conversational level when explicitly instructed to do so (Duffy, 2013; Sapir, 2014). Experimentally, Ho et al. (2000) found that, compared with healthy controls, IWPD overestimated the intensity of their own speech, which the authors suggested may be a potential mechanism for the reduced intensity in hypokinetic dysarthria. Other subtle differences between IWPD and controls regarding loudness perception of self-generated speech include a narrower range of intensity estimation (De Keyser et al., 2016) and a stronger negative effect of background noise on the acuity of intensity estimation (Brajot et al., 2016). In contrast, the aforementioned study by Dromey and Adams (2000) that failed to find a difference between IWPD and controls in loudness perception also applied to loudness perception of their own speech. Results regarding the perception of other acoustic parameters of one’s own speech are also mixed. Abur and Stepp (2020) found no differences between IWPD and controls in auditory acuity for pitch changes in their own voice whereas Mollaei et al. (2019) found that IWPD showed an enhanced ability to identify pitch manipulations externally added to their auditory feedback when actively producing speech but a limited ability for formant discrimination when passively listening to a recording of their own speech. Lastly, Railo et al. (2020) studied speaking-induced suppression in IWPD. Speaking-induced suppression refers to the phenomenon that the amplitude of the 100 ms component in the auditory-evoked response to one’s own voice is reduced during active vocalization than during passive listening (Heinks-Maldonado et al., 2005; Houde et al., 2002). Speaking-induced suppression has been found in both EEG and magnetoencephalographic (MEG) recordings as a decrease of the N1/N100 component amplitude in EEG or of the N100m response amplitude in MEG (Heinks-Maldonado et al., 2005; Houde et al., 2002). Railo et al. (2020) found reduced speaking-induced suppression among IWPD—thus, an atypically large cortical response to self-generated speech sounds.

#### Effects of dopaminergic medications on speech-related sensory impairments in PD

A small number of studies have tested the effects of dopaminergic medications on auditory processing in PD. Pisani et al. (2015) reported that IWPD showed reduced otoacoustic emission response than controls at a subclinical level, but the response increased after dopaminergic treatment. In an EEG study, Gulberti et al. (2015) used a rhythmic click auditory stimulation paradigm with different stimulus rates to test auditory sensory gating in IWPD and examined both the effects of dopaminergic medication in the preoperative stage and the effects of STN-DBS 5 months after surgery; they found that, compared to controls, IWPD showed overall larger AEP amplitudes to clicks at different rates, and neither dopaminergic medication nor STN-DBS had an acute effect, but the AEP amplitude after 5 months of chronic STN-DBS significantly decreased and became closer to that of the controls. In addition, Cameron et al. (2016) investigated how dopaminergic medications affect auditory rhythm discrimination, and found that, compared to the medication OFF state, IWPD in the medication ON state increased sensitivity to changes in simple rhythms but decreased sensitivity to changes in complex rhythms. Finally, to the best of our knowledge, there is no study on the effects of dopaminergic medication on somatosensory processing in the orofacial and laryngeal areas, but there are studies that reported that dopaminergic medications alter somatosensory processing in limb areas on both the perceptual level and the neurophysiological level (Nelson et al., 2012, 2018).

#### Effects of DBS on speech-related sensory impairments in PD

As discussed in the previous section, in their EEG study, Gulberti et al. (2015) reported that 5 months of chronic STN-DBS partially normalized the auditory sensory gating behavior in IWPD but did not find an acute effect of DBS. Two additional MEG studies have reported both acute and long-term effects of STN-DBS on the latency and amplitude of auditory N100m response to pure tone stimuli (Airaksinen et al., 2011; Valkonen et al., 2022).

### Auditory-Motor Perturbation Studies in PD

We conducted a systematic search of the PubMed database to identify studies published up to November 30, 2025, that investigated speech motor control in Parkinson’s disease using feedback-based perturbation paradigms. First, we used the following PubMed query: (“speech”[Title/Abstract] OR “voice”[Title/Abstract] OR “vocal”[Title/Abstract]) AND (“parkinson*”[Title/Abstract] OR “Parkinson’s disease”[Title/Abstract]) AND “feedback”[Title/Abstract] AND (“perturb*”[Title/Abstract] OR “shift*”[Title/Abstract] OR “alter*”[Title/Abstract] OR “manipulat*”[Title/Abstract]) AND 1900/01/01:2025/11/30[Date - Publication]. This yielded 39 articles in PubMed. We read the abstract of each identified article to make sure (1) that the study used an auditory-motor perturbation paradigm as its main experimental method, (2) that the primary outcome measures were compensatory or adaptive changes in the phonatory or articulatory subsystems in conditions with perturbed auditory feedback, and (3) that IWPD were included in the study. All 14 studies that met these criteria are listed in Table 1.

**Table 1.** Auditory-motor perturbation studies on Parkinson’s disease included in the current review.

Reference	Perturbations	Task designs	Additional designs	Patient group	Control group	Compensation or adaptation between groups	Compensation or adaptation between conditions
Kiran and Larson (2001)	*f*_*o*_ shift with magnitude of +100 or −100 cents and duration of 100, 500, or 1000 ms.	– 20 sustained vocalizations for each of the three shift durations. – 1 shift per vocalization.		10 IWPD (on DA medication)	10 age-matched healthy controls	– IWPD showed **similar *f*_*o*_ compensation** as controls.	
Liu et al. (2012)	– *f*_*o*_ shift with magnitude of +100 or –100 cents and duration of 200 ms. – Loudness shifts of +3, −3, +6, or −6 dB, and duration of 200 ms.	– 8 to 13 sustained vocalizations. – 3 to 5 shifts per vocalization.		12 IWPD (off DA medication)	13 healthy controls	IWPD showed **larger *f*_*o*_ and loudness compensations** than controls.	
X. Chen et al. (2013)	*f*_*o*_ shift with magnitude of +50, −50, +100, −100, +200, and −200 cents, and duration of 200 ms.	– 12 sustained vocalizations. – 5 shifts per vocalization.		15 IWPD (off DA medication)	15 age- and sex-matched healthy controls	IWPD showed **larger *f*_*o*_ compensations** than controls.	
Mollaei et al. (2013)	Whole-trial F1 shift with magnitude of +30%.	– Single word productions. – Baseline phase = 30 trials, maximal perturbation phase = 100 trials, ramp-down phase = 40 trials, washout phase = 30 trials.		9 IWPD (off DA medication)	9 age-matched healthy controls	IWPD showed **smaller F1 adaptation** than controls.	
Mollaei et al. (2016)	*For phonatory compensation*: whole-trial *f*_*o*_ shift with magnitude of +50 or +100 cents. *For articulatory compensation*: whole-trial F1 shift with magnitude of +15% or +30%.	*For phonatory compensation*: – 200 trials of prolonged single word production. – *f*_*o*_ shifts on 40 randomly selected trials. *For articulatory compensation*: – 200 trials of prolonged single word production. – F1 shifts on 40 randomly selected trials.		15 IWPD (off DA medication)	15 age- and sex-matched healthy controls	– IWPD showed **larger *f*_*o*_ compensations** than controls. – IWPD showed **smaller F1 compensation** than controls.	
Huang et al. (2016)	*f*_*o*_ shift with magnitude of −200 cents, and duration of 200 ms.	– 25 sustained vocalizations. – 4 shifts per vocalization.	Simultaneous EEG.	18 IWPD (off DA medication)	18 age- and sex-matched healthy controls	IWPD showed **larger *f*_*o*_ compensations** than controls.	
Abur et al. (2018)	Whole-trial *f*_*o*_ shift with magnitude of +100 or −100 cents.	– Trials of sustained vocalization. – Upward perturbation condition, downward perturbation condition, and control condition. – For each condition: baseline phase = 20 trials, ramp phase = 60 trials, maximal perturbation phase = 40 trials, washout phase = 40 trials.	An additional pitch discrimination task was included.	16 IWPD (on DA medication)	19 healthy controls	– IWPD showed **smaller *f*_*o*_ adaptation** than controls on average. – IWPD showed **more variable adaptation response** than controls.	
Huang et al. (2019)	*f*_*o*_ shift with magnitude of −200 cents and duration of 200 ms.	– 25 sustained vocalizations in each condition. – 4 shifts per vocalization.	Internally cued condition vs. externally cued condition (with pitch targets). Simultaneous EEG.	28 IWPD (off DA medication)	28 healthy controls	– In the internally cued condition, IWPD showed **larger *f*_*o*_ compensation** than controls. – In the externally cued condition, IWPD showed **similar *f*_*o*_ compensation** as controls.	IWPD showed **smaller *f*_*o*_ compensation** in the externally cued condition than in the internally cued condition.
Behroozmand et al. (2019)	*f*_*o*_ shift with magnitude of +100 or −100 cents and duration of 200 ms.	– 150 sustained vocalizations in each condition. – 1 shift per vocalization.	STN-DBS ON condition vs STN-DBS OFF condition.	10 IWPD (on DA medication)			IWPD showed **smaller upward *f*_*o*_ compensation** in the DBS ON condition than in the DBS OFF condition.
Mollaei et al. (2019)	*For phonatory compensation*: whole-trial *f*_*o*_ shift with magnitude of +25, +50 or +100 cents. *For articulatory compensation*: whole-trial F1 shift with magnitude of +15% or +30%.	*For phonatory compensation*: – 180 trials of prolonged single word production. – *f*_*o*_ shifts on 90 randomly selected trials. *For articulatory compensation*: – 129 trials of prolonged single word production. – F1 shifts on 60 randomly selected trials.	In the production tasks, after each trial, participants were asked to indicate whether their production had been same or different from the auditory feedback. Furthermore, an additional listening task was included to assess *f*_*o*_ and F1 perceptual discrimination abilities.	15 IWPD (off DA medication)	15 age- and sex-matched healthy controls	– IWPD showed **larger *f*_*o*_ compensations** than controls under +50 and +100 cents perturbations. – IWPD showed **smaller F1 compensation** than controls.	
Bahners et al. (2020)	*f*_*o*_ shift with magnitude of −200 cents and duration of 200 ms.	– 30 sustained vocalizations in each condition. – 6 shifts per vocalization.	STN-DBS ON condition vs. STN-DBS OFF condition. Simultaneous MEG.	20 IWPD (on DA medication)			IWPD showed **similar *f*_*o*_ compensation** in the DBS ON condition and the DBS OFF condition.
Y. Li et al. (2021)	*f*_*o*_ shift with magnitude of −200 cents and duration of 200 ms.	– 25 sustained vocalizations in each condition. – 4 shifts per vocalization.	For IWPD: pre- LSVT® LOUD condition vs. post- LSVT® LOUD condition. Simultaneous EEG.	12 IWPD (off DA medication)	12 age- and sex-matched healthy controls	Pre- LSVT® LOUD, IWPD showed **larger *f*_*o*_ compensation** than controls.	IWPD showed **smaller *f*_*o*_ compensation** post- LSVT® LOUD than pre-LSVT® LOUD.
Abur et al. (2021)	*For phonatory adaptation and compensation*: whole-trial *f*_*o*_ shift with magnitude of +100 cents. *For articulatory adaptation and compensation*: whole-trial F1 shift with magnitude of +30%.	*For phonatory and articulatory adaptations*: – Prolonged single word productions. – Baseline phase = 24 trials, ramp phase = 30 trials, maximal perturbation phase = 30 trials, washout phase = 24 trials. *For phonatory and articulatory compensations*: – 108 trials of prolonged single word productions. – Shifts on 24 randomly selected trials.	A control condition with no *f*_*o*_ shift was included for the phonatory adaptation task. Additional auditory acuity tasks (self-generated *f*_*o*_ and F1) were included.	28 IWPD (on DA medication)	28 age- and sex-matched healthy controls	IWPD showed **similar *f*_*o*_ adaptation, *f*_*o*_ compensation, F1 adaptation, and F1 compensation** as controls.	
Dai et al. (2022)	*f*_*o*_ shift with magnitude of −200 cents and duration of 200 ms.	– 100 sustained vocalizations in each condition. – 2 shifts per vocalization.	For IWPD: active c-TBS over left SMA vs. sham stimulation. Simultaneous EEG.	16 IWPD (off DA medication)	16 age- and sex-matched healthy controls	With sham stimulation, IWPD showed **larger *f*_*o*_ compensation** than controls.	IWPD showed **smaller *f*_*o*_ compensation** with active c-TBS over left SMA than with sham stimulation.

*Note*. c-TBS = continuous theta burst stimulation; DA = dopaminergic; DBS = deep brain stimulation; EEG = electroencephalography; IWPD = individuals with Parkinson’s disease; LSVT® LOUD = Lee Silverman voice treatment; MEG = magnetoencephalography; SMA = supplementary motor area; STN = subthalamic nucleus.

### Auditory-Motor Compensation for Unexpected Perturbations in PD

#### Phonation

Ten studies have been published on the auditory-motor behavior of IWPD in the phonatory subsystem. Most of these studies perturbed *f*_*o*_, but one study also perturbed feedback intensity (Liu et al., 2012). Some of the studies implemented a perturbation in every trial but at random time points during phonation (X. Chen et al., 2013; Dai et al., 2022; Huang et al., 2016, 2019; Kiran & Larson, 2001; Y. Li et al., 2021; Liu et al., 2012), whereas other studies implemented a perturbation on only a randomly selected subset of trials (Abur et al., 2021; Mollaei et al., 2016, 2019). Interestingly, the results of these studies seem to depend on whether the dopaminergic medication state was controlled. On the one hand, studies that tested IWPD in the off-medication state (usually defined as more than 12 hr after the last dose of dopaminergic medication) consistently found that IWPD produced significantly larger vocal compensation than healthy controls (X. Chen et al., 2013; Dai et al., 2022; Huang et al., 2016, 2019; Y. Li et al., 2021; Liu et al., 2012; Mollaei et al., 2016, 2019). On the other hand, studies that did not control for the medication state either reported that IWPD showed similar levels of vocal compensation as healthy controls (Abur et al., 2021) or found that the difference in the compensatory behavior between IWPD and controls was subtle and inconsistent (Kiran & Larson, 2001). In addition, Mollaei et al. (2019) reported that, in addition to their larger *f*_*o*_ compensation, IWPD also showed better ability than controls to identify the *f*_*o*_ perturbations introduced to their auditory feedback, although both groups showed similar pitch discrimination ability in a passive listening task.

It is noteworthy that the larger *f*_*o*_ compensation among IWPD was reduced when an external auditory cue of the *f*_*o*_ target was presented before each trial (Huang et al., 2019), after participants completed an intensive four-week Lee Silverman Voice Treatment (LSVT LOUD) program (Y. Li et al., 2021), or when continuous theta burst stimulation, a form of inhibitory repetitive transcranial magnetic stimulation, was applied over the left supplementary motor area (Dai et al., 2022). Y. Li et al. (2021) reported that this reduction was correlated with the amount of improvement in loudness during passage reading after completing the LSVT LOUD therapy. Moreover, Huang et al. (2016, 2019), Y. Li et al. (2021), and Dai et al. (2022) analyzed AEP in response to the onset of the *f*_*o*_ perturbation and found that IWPD exhibited an enhanced P2 component that accompanied the larger phonatory compensation. Paradoxically, Huang et al. (2019) and Y. Li et al. (2021) reported that this P2 component was further enhanced (rather than reduced) as a result of either the target *f*_*o*_ cueing procedure or LSVT LOUD therapy. The increase in P2 amplitude after external cueing in Y. Li et al. (2021) was significantly correlated with the reduction in phonatory compensation. In contrast, continuous theta burst stimulation over IWPD’s left supplementary motor area reduced P2 amplitude while increasing N1 amplitude (Dai et al., 2022).

#### Articulation

Three studies have been published on how IWPD respond to unexpected auditory perturbations of acoustic parameters associated with the articulatory subsystem. The magnitudes of the unexpected F1 shifts were randomized combinations of 15% upshifts and 30% upshifts in two studies (Mollaei et al., 2016, 2019), whereas the third study implemented 30% F1 upshift as their perturbation magnitude (Abur et al., 2021). However, they produced conflicting results. Mollaei et al. (2016, 2019) found that, compared to healthy controls, IWPD in the off-medication state showed significantly smaller F1 compensation when producing words with the /ε/ vowel sustained over 2.5 s. Abur et al. (2021), however, found no difference in F1 compensation between healthy controls and IWPD in the on-medication state when producing words with the /ɪ/ vowel sustained for 2–3 s. The most critical discrepancy between these studies by Mollaei et al. (2016, 2019) versus Abur et al. (2021) again appears to relate to the medication state. Without dopaminergic medication, IWPD showed atypical compensation to F1 perturbation; with dopaminergic medication, this atypical compensatory behavior did not occur. However, unlike the aforementioned results of increased compensation in the phonatory subsystem in the off-medication state, IWPD show *reduced* compensation in the articulatory subsystem in the off-medication state.

#### Effects of dopaminergic medications on auditory-motor compensation for unexpected perturbations in PD

No study directly tested the effects of dopaminergic medications on auditory-motor compensation. However, as noted above, studies that tested IWPD in the off-medication state consistently found that IWPD produced significantly larger vocal compensation than healthy controls (X. Chen et al., 2013; Dai et al., 2022; Huang et al., 2016, 2019; Y. Li et al., 2021; Liu et al., 2012; Mollaei et al., 2016, 2019), whereas studies that studies that did not control for the medication state either reported that IWPD showed similar levels of vocal compensation as healthy controls (Abur et al., 2021) or found that the difference in the compensatory behavior between IWPD and controls was subtle and inconsistent (Kiran & Larson, 2001).

#### Effects of DBS on auditory-motor compensation for unexpected perturbations in PD

Two studies have examined the effect of STN-DBS on auditory-motor compensation for an *f*_*o*_ perturbation (Bahners et al., 2020; Behroozmand et al., 2019). Both studies tested IWPD in the on-medication state and neither included a control group. Bahners et al. (2020) also recorded MEG data. The findings from the two studies are not consistent. Whereas Behroozmand et al. (2019) reported significant attenuation of IWPD’s vocal compensatory response to a downward *f*_*o*_ perturbation with STN-DBS-ON compared with STN-DBS-OFF (but no such effect for an upward *f*_*o*_ perturbation), Bahners et al. (2020) found no effect of STN-DBS on vocal compensatory behavior. There are no major methodological differences between the studies by Bahners et al. (2020) and Behroozmand et al. (2019) other than the fact that Bahners et al. (2020) included twice as many participants as Behroozmand et al. (2019). Thus, STN-DBS may exert a variable effect (across individuals) on auditory-motor compensation in the phonatory subsystem when *on* dopaminergic medication. Additionally, unlike previous EEG studies revealing that IWPD showed an enhanced P2 component in response to *f*_*o*_ perturbation (Dai et al., 2022; Huang et al., 2016; Y. Li et al., 2021), Bahners et al. (2020) did not find an enhancement of P200m, the MEG equivalent of P2, in response to *f*_*o*_ perturbation during vocalization. Rather, IWPD showed a significant suppression of the P200m in response to the *f*_*o*_ perturbation during vocalization as compared with passive listening, regardless of stimulation state.

### Auditory-Motor Adaptation to Predictable Perturbations in PD

#### Phonation

Two studies have been published on auditory-motor adaptation to consistent *f*_*o*_ perturbations among IWPD, but they produced contradictory results (Abur et al., 2018, 2021). Abur et al. (2018) (15 IWPD, 15 controls) reported that, compared to healthy controls, IWPD as a group adapted significantly less to both 100-cent upward and downward *f*_*o*_ shifts, but Abur et al. (2021) (28 IWPD, 28 controls) found no between-group difference in auditory-motor adaptation to a 100-cent upward *f*_*o*_ shift. Neither of the two studies controlled for medication state, and there appears to be no crucial methodological differences. Hence, the available evidence suggests that PD does not reliably affect auditory-motor adaptation of the phonatory subsystem, but it is unclear if or how this relationship is be modulated by dopaminergic medications.

#### Articulation

We identified two published studies that investigated IWPD’s adaptation to formant perturbations in the auditory feedback. The results are again inconsistent. On the one hand, Mollaei et al. (2013) tested nine IWPD and nine healthy controls and found that IWPD showed reduced adaptation compared to the controls. On the other hand, Abur et al. (2021) found no difference in auditory-motor adaptation to a formant perturbation between 28 IWPD and 28 controls. In addition to the sample size, these two studies differed in two key aspects. First, Mollaei et al. (2013) tested IWPD in their off-medication state, defined as at least 6 hr after their last dopaminergic medication, whereas patients in Abur et al. (2021) were not controlled for their medication state. As discussed above, dopaminergic medication appears to affect phonatory auditory-motor compensation in notable ways, so it is conceivable that it affects auditory-motor adaptation in the articulatory subsystem as well. Further, the adaptation schedules were different between the two studies: Mollaei et al. (2013) abruptly introduced an F1 upshift of 30% (i.e., heard F1 = 130% × produced F1), whereas Abur et al. (2021) gradually introduced an F1 upshift by incrementally increasing it from 0 to 30% over 24 trials. Some studies have suggested that, in limb sensorimotor adaptation, IWPD tend to show typical adaptation with gradual perturbation but reduced adaptation with sudden perturbation (Mongeon et al., 2013; Venkatakrishnan et al., 2011). This might have contributed to the opposing findings between these two speech studies as well.

### Discussion—PD

Table 2 summarizes the results from studies on the effects of PD on speech auditory-motor compensation and adaptation. In sum, current evidence suggests that (1) PD causes exaggerated phonatory compensation, which may be attenuated by dopaminergic medication, and possibly by STN-DBS; (2) PD may cause diminished articulatory compensation, which may be normalized by dopaminergic medication; (3) PD may cause diminished phonatory adaptation; and (4) PD may cause diminished articulatory adaptation, which may be normalized by dopaminergic medication.

**Table 2.** Summary of the number of studies on auditory-motor compensation and adaptation among individuals with Parkinson’s disease, separated by medication state, deep brain stimulation, and reported effects of group or conditions.

	Compensation			Adaptation		
	DA OFF	DA ON or not controlled	DBS	DA OFF	DA ON or not controlled	DBS
Phonation	• PD > control (9)	• PD ≈ control (2)	• ON < OFF (1) • ON ≈ OFF (1)		• PD < control (1) • PD ≈ control (1)	
Articulation	• PD < control (2)	• PD ≈ control (1)		• PD < control (1)	• PD ≈ control (1)	

*Note*. “PD > control” or “PD < control” means that the amount of compensation or adaptation in the Parkinson’s disease group was larger or smaller than that of the control group, respectively, whereas “PD ≈ control” means that the amount of compensation or adaptation in the Parkinson’s disease group did not significantly differ from that in the control group. The numbers in parentheses indicate the number of studies reporting the effect. DA = dopaminergic medication; DBS = deep brain stimulation; PD = Parkinson’s disease.

Exaggerated phonatory compensation in the absence of dopaminergic medication is the best documented finding regarding PD’s effect on feedback control of speech. From a computational perspective, this phenomenon may stem from an increased weighting of (or sensitivity to) the auditory feedback (see Weerathunge, Alzamendi, et al. (2022) and Weerathunge, Tomassi, et al. (2022) for similar computational accounts). In addition to exaggerated *f*_*o*_ compensation, Mollaei et al. (2019) found that IWPD show an enhanced perceptual sensitivity to *f*_*o*_ perturbations in their auditory feedback. Furthermore, neurophysiological studies of auditory processing in PD offer some support for this hypothesis. IWPD were found to show larger frequency following response (Mollaei et al., 2022) and cortical AEP (De Keyser et al., 2021; Lukhanina et al., 2009, 2010) to external auditory stimuli, suggesting greater auditory responsiveness in general. IWPD also showed reduced suppression to their own speech during active vocalization (Railo et al., 2020). This is also consistent with the atypical enhancement of AEP evoked by an *f*_*o*_ perturbation in the auditory feedback (Dai et al., 2022; Huang et al., 2016; Y. Li et al., 2021). At the same time, exaggerated phonatory compensation may also reflect a decrease in the weighting of the somatosensory feedback in PD. Minimizing laryngeal somatosensation via local anesthesia increases phonatory auditory-motor compensatory behavior in typical speakers (Larson et al., 2008). PD is known to be associated with poor somatosensation in the orofacial area (Y.-W. Chen & Watson, 2017; Hammer & Barlow, 2010; Schneider et al., 1986) and with atypical, albeit heightened, perioral reflexes to orofacial stimulation (Caligiuri & Abbs, 1987). Both hypotheses are in line with the notion that PD involves atypical sensory gating caused by basal ganglia pathology (Kaji et al., 2005).

Moreover, the exaggerated phonatory compensation may be a downstream result of a weak feedforward system, which renders the control system overreliant on feedback control and thus more prone to sensory perturbation. In computational models of speech sensorimotor control, the basal ganglia are usually considered more critical for feedforward control, including selecting and initiating appropriate preplanned motor commands (Tourville & Guenther, 2011). A weak feedforward system may explain reduced phonatory adaptation among IWPD (Abur et al., 2018), given that adaptation is thought to depend more on the feedforward control mechanism than the feedback control mechanism. Additionally, X. Chen et al. (2013) and Huang et al. (2016) found that the magnitude of phonatory compensation in PD was correlated with participants’ *f*_*o*_ variability, a finding that can interpreted as suggesting that more phonatory compensation is implemented when feedforward control for the phonatory subsystem is unstable. However, caution is needed when interpreting this correlation given that hypokinetic dysarthria is commonly associated with reduced, not increased, *f*_*o*_ variability (Rusz et al., 2021; Zwirner & Barnes, 1992) and the correlation between speech variability and auditory-motor compensation has been found to be unreliable across studies (Scheerer et al., 2016; Scheerer & Jones, 2012). It should be noted that the different mechanisms postulated above are not mutually exclusive and that they may interact to produce the observed exaggerated phonatory compensation.

Dopaminergic medication appears to restore the exaggerated phonatory auditory-motor compensation in PD to a more typical level, implying a role for the dopaminergic pathways in speech sensorimotor control. Nevertheless, it should be noted that this effect of dopaminergic medications is inferred from comparing the studies that tested IWPD in the dopaminergic medication OFF state and the studies that did not control for dopaminergic medication, as no auditory-motor compensation studies directly tested the effects of dopaminergic medication. Future studies that directly compare auditory-motor compensation and adaptation between the on-medication and the off-medication states within the same group of IWPD are needed to further confirm the role of dopamine. STN-DBS, on the other hand, appears to be less effective in affecting auditory-motor compensation in the phonatory subsystem, as the only two existing studies have produced contradictory results (Bahners et al., 2020; Behroozmand et al., 2019). Neither study controlled for dopaminergic medication, which might have interacted with the effect of STN-DBS. Moreover, the effects of STN-DBS on speech sensorimotor control may be confounded by the involvement of neighboring white matter tracts such as the dentatorubrothalamic tract on an individual level (Fenoy et al., 2017). It is also possible that STN-DBS may affect the speech sensorimotor control system through neuronal reorganization induced by long-term stimulation, and therefore its effects may not be revealed in an acute manner (Ashkan et al., 2017). Such long-term neuronal reorganization may explain the difference in neurophysiological findings between Bahners et al. (2020) and Huang et al. (2016). Future studies on the effects of the STN-DBS on the speech sensorimotor control system will need to take these factors into account.

Intriguingly, it was found that both external auditory cueing and the LSVT LOUD therapy attenuated the exaggerated magnitude of phonatory compensation in PD, but paradoxically further increased the P2 component in AEP to an *f*_*o*_ perturbation in the auditory feedback (Huang et al., 2019; Y. Li et al., 2021). Both external cueing and the LSVT LOUD are well-attested therapeutic methods that improve phonation in hypokinetic dysarthria, especially overall speech intensity (Goberman & Elmer, 2005; Ho et al., 1999; Martel Sauvageau et al., 2015; Nakayama et al., 2020; Ramig et al., 2001; Tjaden et al., 2013). Both methods work by making IWPD constantly aware of their speech goal, either through external cues or through explicit instructions (“Think LOUD!”). Therefore, such explicit strategies might exert higher cognitive influence over the impaired speech sensorimotor control system in PD and modulate (normalize) auditory-motor compensatory behavior. This modulation may underlie improvements in motor speech symptoms as well, as shown by their positive correlation (Y. Li et al., 2021). Such top-down cognitive control of the speech system may also explain the paradoxical post-therapy P2 increase when hearing *f*_*o*_ perturbations, as this response may originate from areas that have been shown to be modulated by the LSVT LOUD therapy (e.g., dorsolateral prefrontal cortex) (Narayana et al., 2010).

Finally, it appears that PD-related sensorimotor control differences in the articulatory subsystem are distinct from those in the phonatory subsystem. Without dopaminergic medication, IWPD exhibited diminished, not exaggerated, articulatory compensation (Mollaei et al., 2016) and adaptation (Mollaei et al., 2013), and dopaminergic medication seems to normalize these compensatory and adaptive responses (Abur et al., 2021). From a computational perspective, these findings can be suggested to reflect a decrease in the sensitivity to auditory feedback errors in the articulatory sensorimotor control system, leading to reduced updating of future motor commands. However, two important caveats should be noted. First, compared to the phonatory system, the number of studies on articulatory compensation and adaptation are too small to verify the robustness of these atypical behaviors. Second, although the relation between error-based updates of the feedforward control system (adaptation) and the online feedback control system (compensation) may appear intuitive in theory, data from typical speakers have shown no correlation between auditory-motor compensation and adaptation— thus, they may be driven by independent processes (Daliri, 2021; Raharjo et al., 2021). Nonetheless, the available data so far suggest that sensorimotor control of the phonatory subsystem and the articulatory subsystem are distinct and that they are affected in different ways in PD patients. The relation between the phonatory and the articulatory sensorimotor control systems is an underexplored topic. According to one study, typical speakers can compensate for *f*_*o*_ and formant perturbations at the same time, and phonatory compensation tends to be larger than articulatory compensation (Xu et al., 2020). Further, typical speakers show opposite patterns of compensation and adaptation test–retest reliability in the two subsystems: phonatory compensation is more reliable than phonatory adaptation, whereas articulatory adaptation is more reliable than articulatory compensation (Kapsner-Smith et al., 2024). These results suggest that the articulatory subsystem may be inherently less reliant on online auditory feedback than the phonatory subsystem, which is also supported by the observation that post-lingual deafness causes more rapid degradation of phonation than articulation (Cowie & Douglas-Cowie, 1992). This difference in feedback sensitivity between phonation and articulation may be exacerbated by PD. Indeed, Mollaei et al. (2019) found that IWPD performed worse than the controls in passive formant discrimination, but better than controls in identifying pitch perturbations during active vocalization. In addition, the authors of Mollaei et al. (2016) speculated that PD may uniquely limit feedforward control of articulators due to impaired nigrostriatal dopaminergic projections to frontal tongue motor regions (Ridding et al., 1995; Sabatini et al., 2000). More studies are needed to untangle the relationship between phonatory and articulatory sensorimotor control systems in both typical speakers and IWPD.

To sum up, although the exaggerating effect of PD on auditory-motor compensation in the phonatory subsystem has been found in a number of studies, the effects of PD on other aspects of phonatory and articulatory sensorimotor control remains largely unclear and needs further study. Important topics yet to be fully elucidated include the effects of PD on phonatory adaptation, articulatory compensation, and articulatory adaptation, as well as more rigorous tests of the effects of dopaminergic medication and STN-DBS on these behaviors.

## DYSTONIA

### Motor Speech Symptoms in Dystonia

Dystonia is a condition characterized by sustained or repeated involuntary muscle contractions that produce atypical postures and repetitive movements (Steeves et al., 2012). The etiology of most cases of dystonia remains unknown (Balint et al., 2018), but there is strong evidence that basal ganglia circuits play a critical role given that dystonia can result from lesions to the basal ganglia (Bhatia & Marsden, 1994) and atypical electrophysiological activity in the basal ganglia has been observed in patients with dystonia (Brittain & Brown, 2014; Neumann et al., 2017). Dystonia symptoms can occur in various parts of the body, including structures involved in speech production such as the larynx and orofacial area. The motor speech disorders resulting from dystonias in these structures are considered subtypes of hyperkinetic dysarthria (Duffy, 2013). Among these, laryngeal dystonia (LD)—also known as spastic or spasmodic dysphonia—is an isolated, focal dystonia that causes sustained or repeated involuntary contraction of the laryngeal musculatures that result in phonatory impairments (Simonyan et al., 2021). The current review focuses on LD, because its effect on speech production is the most well-studied in dystonia and results from auditory-motor perturbation studies are available for LD.

LD can be further categorized as adductor LD, abductor LD, and mixed LD, and is usually triggered specifically by speaking (Duffy, 2013; Simonyan et al., 2021). Adductor LD, which accounts for approximately 90% of LD cases, is characterized by a strained, groaning, and effortful voice quality and uncontrolled vocal breaks during speech, caused by spasms of the laryngeal adductor muscles leading to vocal fold hyperadduction (Duffy, 2013). Acoustic features of adductor LD include frequent phonatory breaks in the speech signal, frequent aperiodic segments, large standard deviation of *f*_*o*_, and poor voice quality measures (Z. Chen et al., 2019; Cimino-Knight & Sapienza, 2001; Roy et al., 2008, 2014; Sapienza et al., 1998, 1999, 2000, 2002; Zwirner et al., 1993). The symptoms and severity of adductor LD can vary greatly depending on the specific task, such as sustained vowel productions or connected speech (Roy et al., 2005, 2014; Sapienza et al., 1999, 2000). Abductor LD is caused by spasms in the abductor laryngeal muscles that result in an intermittent or near-constant breathy or aphonic voice quality (Duffy, 2013; Merson & Ginsberg, 1979). Acoustically, abductor LD also shows frequent phonatory breaks and aperiodic segments and high *f*_*o*_ variability (Edgar et al., 2001), but it can be differentiated from adductor LD by the existence of fricative-like noises during phonation (Wolfe & Bacon, 1976) and prolonged voice onset time for voiceless consonants (Cannito et al., 2014; Edgar et al., 2001; Yanagida et al., 2015).

#### Effects of botulinum toxin injections on motor speech symptoms in LD

The most common treatment for LD is botulinum toxin (Botox) injections in the laryngeal muscles. Botox injections result in temporary paresis or paralysis of the dystonic muscles due to inhibition of acetylcholine release in the neuromuscular junction (van Esch et al., 2017). For most individuals, Botox injections lead to improved self-ratings of vocal function, relieve of symptoms, and better acoustic measurements such as a reduced number of phonatory breaks and aperiodic segments, decreased *f*_*o*_ variability, and more typical voice quality (Sanuki, 2023; van Esch et al., 2017).

#### Effects of DBS on motor speech symptoms in LD

Several case reports and small-scale clinical trials have been published on the effects of DBS on LD. Overall, preliminary evidence suggests that DBS of the GPi and the Vim nucleus of thalamus can improve self-reported voice-related quality of life and perceptual ratings of LD in some individuals, but larger and more rigorous studies are needed (Evidente et al., 2020; Honey et al., 2021; Krüger et al., 2020; Patel et al., 2022; Poologaindran et al., 2018).

### Speech-Related Sensory Impairments in Dystonia

Like PD, dystonia is known to be associated with sensory processing deficits due to basal ganglia pathology (Kaji et al., 2005), such as atypical temporal-spatial discrimination of somatosensory stimuli (Tinazzi et al., 2009). It is often observed that sensory “tricks” applied to the affected structures can temporarily alleviate the symptoms of various types of dystonia, including LD (Ramos et al., 2014; Sanuki, 2023). However, few studies directly examined speech-related sensory processing in individuals with LD (IWLD). Khosravani et al. (2019) found that vibrato-tactile stimulation of the skin in the laryngeal area leads to short-term abatement of LD symptoms and yields changes in EEG-recorded neural oscillations over somatosensory-motor cortex that are similar to those induced by sensory “tricks” for other types of dystonia. Deleyiannis et al. (1999) and Ludlow et al. (1995) found that IWLD showed disinhibited electromyographic responses in the thyroarytenoid muscles to successive electric stimulations of the superior laryngeal nerve. To date, no behavioral differences in auditory processing have been reported for IWLD but several studies have found that a subset of IWLD exhibited atypical auditory brainstem response components with larger amplitudes and longer latencies (Finitzo-Hieber et al., 1982; Schaefer et al., 1983, 1985).

### Auditory-Motor Perturbation Studies in Dystonia

For a systematic search of studies published before November 30, 2025, that used auditory-motor perturbation paradigms with IWLD, we used the following PubMed query: (“speech”[Title/Abstract] OR “voice”[Title/Abstract] OR “vocal”[Title/Abstract]) AND (“dystonia”[Title/Abstract] OR “spasmodic dysphonia”[Title/Abstract]) AND “feedback”[Title/Abstract] AND (“perturb*”[Title/Abstract] OR “shift*”[Title/Abstract] OR “alter*”[Title/Abstract] OR “manipulat*”[Title/Abstract]) AND 1900/01/01:2025/11/30[Date - Publication]. This yielded 8 articles. Among these, 2 articles met the criteria mentioned in this section and are included in this review (Table 3).

**Table 3.** Auditory-motor perturbation studies on laryngeal dystonia included in the current review.

Reference	Perturbations	Task designs	Additional design	Patient group	Control group	Compensation or adaptation between groups	Compensation or adaptation between conditions
Thomas et al. (2021)	*f*_*o*_ shift with magnitude of +200 or −200 cents and duration of 200 ms.	– 120 to 170 trials of sustained vocalizations. – 1 shift on 70% to 80% of trials.		15 Individuals with ADLD (Botox injection not controlled)	11 age-matched controls	IWLD showed **larger *f*_*o*_ compensation** than controls.	
Kothare et al. (2022)	*f*_*o*_ shift with magnitude of +100 and −100 cents and duration of 400 ms.	– 120 trials of sustained vocalization. – 1 shift per vocalization.	Simultaneous MEG.	22 Individuals with ADLD (3 months after the previous Botox injection)	18 age-matched healthy controls	IWLD showed **similar *f*_*o*_ compensation** as controls.	

*Note*. ADLD = adductor laryngeal dystonia; IWLD = individuals with laryngeal dystonia; MEG = magnetoencephalography.

### Auditory-Motor Compensation for Unexpected Perturbations in Dystonia

#### Phonation

There are two published studies on the effects of LD on auditory-motor compensation under unexpected *f*_*o*_ perturbations (Kothare et al., 2022; Thomas et al., 2021). The results of those two studies are not in agreement. Thomas et al. (2021) (15 individuals with adductor LD, 11 healthy controls) found that individuals with adductor LD exhibited significantly larger compensation than healthy controls whereas Kothare et al. (2022) (22 individuals with adductor LD, 18 healthy controls) reported no significant difference in *f*_*o*_ compensation between the adductor LD group and the control group. Moreover, Thomas et al. (2021) found a negative correlation between compensation magnitude and a subjective rating of adductor LD severity whereas Kothare et al. (2022) reported a positive correlation between compensation magnitude and an acoustic measure of LD severity. There are several methodological differences that may have contributed to the discrepant results, including the magnitude of the applied perturbation (±200 cents in Thomas et al. (2021) vs. ±100 cents in Kothare et al. (2022)). However, the most notable difference is the inclusion criterion regarding Botox injection. Thomas et al. (2021) tested individuals with adductor LD during their clinical visit to receive the Botox injection and did not control for whether they had been injected or not, whereas Kothare et al. (2022) only included individuals who had received Botox injection at least 3 months prior to the study. Despite finding no between-group behavioral differences, Kothare et al. (2022) reported that individuals with adductor LD exhibited atypical MEG activity in premotor, motor, somatosensory and auditory cortices after onset of the *f*_*o*_ perturbation.

### Auditory-Motor Adaptation to Predictable Perturbations in Dystonia

To date, no studies have been published on auditory-motor adaptation in LD.

### Discussion—Dystonia

Table 4 summarizes the results from available studies on the effects of LD on phonatory auditory-motor compensation. Only two studies have been published so far, with conflicting results. Thus, at this time, it is difficult to draw conclusions about how LD affects the speech sensorimotor control system. However, this area of research deserves more attention. Just like PD, dystonia is primarily associated with basal ganglia pathology that can lead to atypical sensory gating mechanisms (Kaji et al., 2005). Furthermore, LD is associated with certain somatosensory and auditory processing deficits similar to PD. Therefore, the exaggerated phonatory compensation found by Thomas et al. (2021) may point to further similarities between LD and PD in terms of phonatory sensorimotor control, potentially driven by similar somatosensory processing deficits shared by both disorders. In addition, similar to the enhanced phonatory compensation found by Thomas et al. (2021), a recent study reported that IWLD showed more pronounced *f*_*o*_ centering than controls (Rangwala et al., 2026), a phenomenon that reflects auditory feedback-based correction in natural speech (Niziolek et al., 2013). On the other hand, the null results from Kothare et al. (2022), who controlled for the time of most recent Botox injection, complicates this hypothesis. To gain a deeper and more comprehensive understanding of the sensorimotor control mechanisms in LD and how they differ from PD, additional auditory-motor perturbation paradigms, such as compensation in response to formant perturbations as well as adaptation to either *f*_*o*_ or formant perturbations, should be conducted. In addition, future studies on LD should also pair such behavioral paradigms with neurophysiological measures like EEG or MEG, especially given that IWPD have been found to show atypical speech-related neurophysiological activity, such as reduced speaking-induced suppression and enhanced auditory response to *f*_*o*_ perturbation (Dai et al., 2022; Huang et al., 2016; Y. Li et al., 2021; Railo et al., 2020). Such neurophysiological studies on speech sensorimotor processing and integration in LD will add to the growing number of studies that have found structural and functional differences in the auditory and somatosensory cortices between IWLD and controls, including increased gray matter volume and cortical thickness of laryngeal sensorimotor cortex and auditory cortex (Simonyan & Ludlow, 2012), increased cortical surface area of primary somatosensory cortex (Kostic et al., 2016), reduced functional connectivity in left premotor/somatosensory cortices (Putzel et al., 2018), atypical blood-oxygen-level-dependent response in somatosensory and auditory areas during vocalization (increase: Daliri et al. (2020), Kiyuna et al. (2014), Simonyan and Ludlow (2010, 2012); decrease: Haslinger et al. (2005)) and voice perception (increased: Kanazawa et al. (2020)).

**Table 4.** Summary of the number of studies on phonatory auditory-motor compensation among individuals with laryngeal dystonia, separated by medication state and reported effects of group or conditions.

	Compensation	
	Botox OFF	Botox ON or not controlled
Phonation	• LD ≈ control (1)	• LD > control (1)

*Note*. Same notations as Table 2. LD = laryngeal dystonia.

## CEREBELLAR DEGENERATION

### Motor Speech Symptoms in CD

Cerebellar degeneration (CD) is an umbrella term for a diverse array of diseases including various hereditary or sporadic types of spinocerebellar ataxia, Friedreich’s ataxia, and the cerebellar subtype of multiple system atrophy. Individuals with CD (IWCD) usually share many distinctive symptoms such as broad-based gait, ocular instability, dysmetria, intention tremor, and ataxic dysarthria (Bodranghien et al., 2016). In addition, many recent studies have found that cerebellar degeneration plays a primary role in the pathophysiology of essential tremor (Louis, 2018; Louis & Faust, 2020a, 2020b; Pan & Kuo, 2022). Essential tremor (ET) is one of the most common movement disorders and is characterized by postural and kinetic tremor of the hands or limbs in the frequency range of 4–12 Hz (Louis & McCreary, 2021). Therefore, in this review, we classify ET as a type of cerebellar degeneration. However, due to the distinct nature of motor speech symptoms in most other types of CD versus ET, they are discussed here separately.

The constellation of motor speech symptoms associated with most types of CD is referred to as ataxic dysarthria (Darley et al., 1969; Duffy, 2013). *Phonatory* impairments of ataxic dysarthria have been described as “vocal instability” (Duffy, 2013). Perceptually, it is characterized by excessive variations of loudness and pitch and unstable voice quality (Ackermann & Ziegler, 1994; Darley et al., 1969; Duffy, 2013). Some studies have also identified vocal tremor in approximately 50% of individuals with ataxic dysarthria of varying etiologies (Boutsen et al., 2011; Hlavnička et al., 2020). Acoustically, ataxic dysarthria has been related to excessive variations in *f*_*o*_ and intensity (Ackermann & Ziegler, 1994; Boutsen et al., 2011; Gentil, 1990; Kent et al., 1997, 2000; Noffs et al., 2020; Schalling et al., 2007) and poor voice quality measures (Ackermann & Ziegler, 1994; Boutsen et al., 2011; Gentil, 1990; Jannetts & Lowit, 2014; Kent et al., 1997, 2000; Noffs et al., 2020; Schalling et al., 2007; Vogel et al., 2017).

Perceptually, the main *articulatory* feature of ataxic dysarthria is irregular articulatory breakdowns, including distorted vowels, irregular diadochokinesis rate, and discoordination between speaking and breathing (Duffy, 2013). This may be a manifestation of timing deficits in movement coordination, a characteristic of cerebellar degeneration (Manto, 2018). Many acoustic studies have confirmed these speech timing and coordination deficits in individuals with ataxic dysarthria and found that IWCD show slower repetition rate and irregular rhythm in speech diadochokinesis tasks (Ackermann et al., 1995; Gentil, 1990; Kent et al., 2000; Portnoy & Aronson, 1982; Tanaka et al., 2021; Ziegler & Wessel, 1996). Rhythmic irregularity was observed in speech kinematics and muscle activity as well (Hirose et al., 1978). Further evidence for a problem with the temporal aspects of speech includes atypical voice-onset time and vowel duration in ataxic speech (Ackermann et al., 1999b; Ackermann & Hertrich, 1997; Tykalova et al., 2017).

For individuals with ET (IWET), the most common motor speech symptom is essential voice tremor, or tremor in the laryngeal musculature. Perceptually, essential voice tremor is characterized by involuntary rhythmic modulation of vocal pitch and loudness (Duffy, 2013). Acoustically, it corresponds to periodic fluctuations of *f*_*o*_ and the amplitude of the speech signal. The tremor frequency in essential voice tremor can range from 1.5 Hz to 6 Hz (Hlavnička et al., 2020), but the most common range is 5–6 Hz (Duffy, 2013). Essential voice tremor frequency is also modulated by *f*_*o*_ and loudness, and higher *f*_*o*_ and greater loudness are associated with higher essential voice tremor frequency for both *f*_*o*_ and loudness (Dromey et al., 2002). Tremor has also been observed directly with laryngeal electromyography and laryngoscopy (Gamboa et al., 1998; Gillivan-Murphy & Miller, 2011; Koda & Ludlow, 1992). Besides vocal tremor, the speech of some IWET also exhibits other characteristic acoustic features, including poor voice quality and a reduced *f*_*o*_ and intensity range (Akkunje et al., 2021; Gamboa et al., 1998).

#### Effects of DBS on motor speech symptoms in ET

Although DBS is not a common treatment for most types of CD, it is a common procedure for pharmacologically refractory ET, with the most well-established stimulation target being the Vim nucleus of the thalamus (Deuschl et al., 2011; Milosevic et al., 2018; Papavassiliou et al., 2004). Sites in the posterior subthalamic area (PSA), especially the caudal zona incerta, have also been selected as alternative DBS targets but their benefits over Vim-DBS remain inconclusive (Eisinger et al., 2018; Xie et al., 2012). Regardless of the exact stimulation target, studies on the effects of DBS on speech production in ET have focused on two distinct phenomena: (1) effect of DBS on reducing essential voice tremor; and (2) adverse speech outcomes caused by DBS, known as stimulation-induced dysarthria.

Several studies have been published on the effects of Vim-DBS and PSA-DBS on essential voice tremor. Overall, most studies reported significant reduction of voice tremor severity, at the group level, with DBS-ON as compared with pre-DBS or DBS-OFF. However, large individual variability in essential voice tremor reduction was reported in some studies (Carpenter et al., 1998; Hägglund et al., 2016). There appears to be little difference in the outcomes between bilateral and unilateral stimulation (Avecillas-Chasin et al., 2018; Kundu et al., 2018; Sandström et al., 2018), but individuals differ in their “dominant” side for essential voice tremor reduction (Avecillas-Chasin et al., 2018). Additionally, several studies have investigated the optimal stimulating targets in either Vim or PSA for essential voice tremor, but so far there is great variability in the findings (Avecillas-Chasin et al., 2018; Erickson-DiRenzo et al., 2020; Kundu et al., 2018; Sandström et al., 2019).

Interestingly, many studies have reported speech impairments as a result of DBS in IWET, especially among individuals with no preoperative speech complaints (Alomar et al., 2017). In a systematic review on stimulation-induced dysarthria, Alomar et al. (2017) found that, among the 19 included studies, 20.4% of IWET patients reported speech impairment post-DBS. Although most of these reports were based on self-report or perceptual scores, an increasing number of studies have attempted to use acoustic and kinematic measurements to quantify speech outcomes of DBS among IWET without preoperative essential voice tremor. Although some individuals showed minimal acoustic or aerodynamic changes related to the phonatory subsystem after Vim-DBS (Ruckart et al., 2020), many studies reported speech deterioration as compared with the preoperative state or the DBS-OFF state. Both Vim-DBS and PSA-DBS have been found to lead to lower speech diadochokinesis rate, presence of voicing during voiceless consonants, and reduced distinction between consonants and vowels in their intensity envelope (Barbe et al., 2014; Becker et al., 2017, 2020; Mücke et al., 2014; Petry-Schmelzer et al., 2021). These deficits primarily reflect impaired coordination both between the articulators and between the phonatory and the articulatory subsystems. Articulographic measurements also showed that Vim-DBS aggravated dyscoordination in labial and lingual movements (Hermes et al., 2019; Mücke et al., 2018). These articulatory coordination deficits are reminiscent of ataxic dysarthria in CD. This similarity may relate to the fact that Vim receives cerebellar information in the cortico-cerebello-thalamo-cortical circuit through the dentatorubrothalamic tract. Therefore, electrical stimulation at Vim or nearby areas such as the PSA may result in effects that resemble microlesions of the cortico-cerebello-thalamo-cortical pathways (Groppa et al., 2014; Mücke et al., 2018; Petry-Schmelzer et al., 2021), such as ataxic dysarthria (Kent et al., 2001; von Cramon, 1981). As discussed in a previous section, the recruitment of the dentatorubrothalamic tract by STN-DBS has also been associated with worsened speech outcomes in PD (Fenoy et al., 2017). In addition, damage to the corticobulbar tract in the internal capsule has also been proposed as a contributing factor to stimulation-induced dysarthria in ET (Becker et al., 2017), given the proximity of Vim to the internal capsule (Yamada et al., 2010). A recent study found that 62.4% of the variance of stimulation-induced dysarthria caused by Vim- and PSA-DBS can be explained by the stimulation affecting tracts connecting with the ipsilateral precentral gyrus, either hemispheres of the cerebellum, or the ipsilateral brain stem (Petry-Schmelzer et al., 2021).

### Speech-Related Sensory Impairments in CD

Auditory processing has been studied in several subtypes of CD. In Friedreich’s ataxia, pure-tone audiometry results appear within typical limits, but many affected individuals exhibit atypical auditory brainstem response (Amantini et al., 1984; Cassandro et al., 1986; Rance et al., 2008, 2010) and poor performance for spectrotemporal processing and speech recognition in noise (Quine et al., 1984; Rance et al., 2008, 2010). Axonal degeneration of sensory nerves may underlie these auditory deficits in Friedreich’s ataxia (Hughes et al., 1968; Nolano et al., 2001). A few additional studies have looked at auditory processing and speech perception in other types of CD. Individuals with different types of spinocerebellar ataxia have been found to show a wide range of audiometric and electrophysiological differences (Zeigelboim et al., 2013).

Given the presence of problems with temporal aspects of speech in ataxic dysarthria, several studies have examined auditory temporal processing in CD. Ackermann et al. (1997, 1999b) reported that IWCD showed poorer performance in categorical perception tasks based on temporal speech features such as voice onset time and vowel length. Further, Ackermann et al. (1999a) reported general auditory temporal processing deficits among individuals with ataxia due to degenerative cerebellar atrophy. In addition, IWCD were found to show deficits in the perception of duration-based absolute timing but not of beat-based relative timing (Grube et al., 2010). Together, these findings suggest a domain general temporal processing deficit in CD.

No studies were found on auditory processing in IWET. Interestingly, due to the oscillatory nature of voice tremor, it has been speculated that delay in the processing of sensory feedback, either auditory or somatosensory, may play a role in generating and sustaining the tremor based on a closed-loop model of vocal motor control (Leydon et al., 2003). Experiments with altered auditory feedback in typical speakers have resulted in tremor-like vocal modulations (Leydon et al., 2003), and computational models have proposed to simulate the “tremor-generating” mechanisms (Brajot & Neiman, 2020). Regarding somatosensory processing, Conte et al. (2015) found that individuals with isolated voice tremor—but not individuals with upper limb tremor—showed elevated somatosensory temporal discrimination thresholds in the hand, neck, and face. Similar somatosensory temporal discrimination deficits have also been found in PD and dystonia (Artieda et al., 1992; Conte et al., 2013; Tinazzi et al., 2013), which led the authors to speculate about basal ganglia involvement in the etiology of isolated voice tremor (Conte et al., 2015).

### Auditory-Motor Perturbation Studies in CD

For a systematic search of studies published before November 30, 2025, that used auditory-motor perturbation paradigms with IWCD, we used the following PubMed query: (“speech”[Title/Abstract] OR “voice”[Title/Abstract] OR “vocal”[Title/Abstract]) AND (“cerebellar degeneration”[Title/Abstract] OR “ataxia”[Title/Abstract] OR “essential tremor”[Title/Abstract]) AND “feedback”[Title/Abstract] AND (“perturb*”[Title/Abstract] OR “shift*”[Title/Abstract] OR “alter*”[Title/Abstract] OR “manipulat*”Title/Abstract]) AND 1900/01/01:2025/11/30[Date - Publication]. This yielded 11 articles. Six articles met the criteria mentioned in this section. Two more recent studies were included based on suggestions received during the peer-review process. In total, eight articles are included in this review (Table 5). Note that no IWET were included in the eight studies identified; the participants included in these studies were predominantly patients with various types of spinocerebellar ataxia or idiopathic cerebellar degeneration.

**Table 5.** Auditory-motor perturbation studies on cerebellar degeneration included in the current review.

Reference	Perturbations	Task designs	Additional designs	Patient group	Control group	Compensation or adaptation between groups	Compensation or adaptation between conditions
Parrell et al. (2017)	*For articulatory adaptation*: whole-trial F1 shift with magnitude of −150 Hz. *For articulatory compensation*: whole-trial F1 shifts with magnitude of +150 Hz or −150 Hz.	*For articulatory adaptation (2 conditions)*: – Single word productions. – Gradual perturbation condition: baseline phase = 30 trials, ramp-up phase = 30 trials, maximal perturbation phase = 60, ramp-down phase = 30 trials, washout phase = 20 trials. – Sudden perturbation condition: baseline phase = 30 trials, maximal perturbation phase = 90 trials, washout phase = 50 trials. *For articulatory compensation*: – 170 trials of prolonged single word productions. – F1 shifts on 60 or 80 randomly selected trials.		19 IWCD (SCA2, 3, 5, 6, 7, 8, unknown CD)	14 age-matched controls	– IWCD showed **smaller F1 adaptation** than controls. – IWCD showed **larger F1 compensation** than controls.	IWCD showed **similar F1 adaptation** in the gradual perturbation condition and the sudden perturbation condition.
Houde et al. (2019)	*f*_*o*_ shift with magnitude of +100 cents or −100 cents and duration of 400 ms.	– 74 sustained vocalizations. – 1 shift per vocalization.		16 IWCD (SCA2, 3, 5, 6, 7, unknown CD)	11 age-matched healthy controls	IWCD showed **larger *f*_*o*_ compensation** than controls.	
W. Li et al. (2019)	*f*_*o*_ shift with magnitude of −200 cents or −500 cents and duration of 200 ms.	– 40 sustained vocalizations. – 5 shifts per vocalization.	Simultaneous EEG.	12 IWCD (SCA1, 3)	12 age- and sex-matched healthy controls	IWCD showed **larger *f*_*o*_ compensation** than controls.	
Q. Lin et al. (2022)	*f*_*o*_ shift with magnitude of +200 cents or −200 cents and duration of 200 ms.	– 100 sustained vocalizations. – 2 shifts per vocalization.	Active vs. sham c-TBS over the right cerebellum. Simultaneous EEG.	19 IWCD (SCA1, 2, 3, 6)			IWCD showed **smaller *f*_*o*_ compensation** under active c-TBS over the right cerebellum than under sham stimulation.
Parrell et al. (2021)	*For articulatory adaptation*: whole-trial F1 shift with magnitude of −125 mels. *For articulatory compensation*: whole-trial F1 shift with magnitude of +125 mels or −125 mels.	*For articulatory adaptation*: – Single word productions. – Baseline phase = 60 trials, perturbation phase = 100 trials, washout phase = 30 trials. *For articulatory compensation*: – 120 trials of prolonged single word production. – F1 shifts on 60 randomly selected trials.	Two additional visuomotor rotation reaching tasks were also tested.	22 IWCD (AOA2, SCA3, 6, 8, unknown CD)	15 age-matched healthy controls	– IWCD showed **smaller F1 adaptation** than controls. – IWCD showed **similar F1 compensation** as controls.	
Hilger et al. (2025)	*f*_*o*_ shift with magnitude of +200 cents or −200 cents and duration of 200 ms.	– 90 sustained vocalizations. – 2 shifts per vocalization.	Additional sentence production tasks with *f*_*o*_ perturbations and different semantic focus patterns.	22 IWCD (AOA2, FA, Gulten Ataxia, SCA1, 2, 3, 5, 6, 7, 15, SCA-unknown, SCAR8)	29 age-matched healthy controls	IWCD showed **larger *f*_*o*_ compensation** than controls.	
Slis and Parrell (2026)	*For phonatory adaptation*: Experiment 1: whole-trial *f*_*o*_ shift with magnitude of −100 cents. *For phonatory compensation*: Experiment 2: *f*_*o*_ shift with magnitude of +100 cents or −100 cents starting 200–500 ms after vocalization onset and continuing throughout the trial; Experiment 3: *f*_*o*_ shift with magnitude of +100 cents or −100 cents starting from vocalization onset and continuing for 400–550 ms; Experiment 4: *f*_*o*_ shift with magnitude of +100 cents or −100 cents starting 200–500 ms after vocalization onset and continuing for 400 ms.	*For phonatory adaptation*: – Single word production and sustained vocalization (separate tasks). – Baseline phase = 20 trials, maximal perturbation phase = 40 trials, washout phase = 20 trials. *For phonatory compensation*: – 105 trials of sustained vocalizations. – Shifts on 80 randomly selected trials.	Two control tasks (word production and sustained vocalization) with no *f*_*o*_ shift were included for the phonatory adaptation task.	43 IWCD (AOA2, CANVAS, idiopathic, SCA1, 2, 3, 4, 5, 6, 8, 13, 14, 16, 27, tumor)	48 age-matched healthy controls	– IWCD showed **no *f*_*o*_ adaptation** as controls in either word production or sustained vocalization. – IWCD showed overall **similar *f*_*o*_ compensation** as controls, with subtle group differences depending on the timing of perturbation implementation and measurement window.	
Karlin and Parrell (2026)	Vowel duration increase with magnitude of +60 ms.	– Single word productions (the word “best”). – Baseline phase = 20 trials, ramp phase = 20 trials, hold phase = 30 trials, washout phase = 20 trials.	An additional perceptual acuity task of vowel duration was included.	34 IWCD (AOA2, CANVAS, idiopathic, SCA1, 2, 3, 4, 5, 6, 8, 14, tumor)	34 age-matched healthy controls	– IWCD showed **no vowel duration adaptation** as controls.	

*Note*. AOA = ataxia with oculomotor apraxia; CANVAS = cerebellar ataxia, neuropathy, vestibular areflexia syndrome; c-TBS = continuous theta burst stimulation; EEG = electroencephalography; FA = Friedreich’s ataxia; IWCD = individuals with cerebellar degeneration; SCA = spinocerebellar ataxia; SCAR = spinocerebellar ataxia recessive autosomal.

### Auditory-Motor Compensation for Unexpected Perturbations in CD

#### Phonation

There are five studies on how CD affects phonatory compensation to unexpected *f*_*o*_ perturbations, four studies compared IWCD with healthy control participants (Hilger et al., 2025; Houde et al., 2019; W. Li et al., 2019; Slis & Parrell, 2026) and one study assessed the modulating effect of cerebellar continuous theta burst stimulation in IWCD (Q. Lin et al., 2022). Four out of the five studies showed that IWCD exhibited enhanced phonatory compensation as compared with healthy controls (Hilger et al., 2025; Houde et al., 2019; W. Li et al., 2019; Q. Lin et al., 2022), despite the heterogeneity of CD diagnoses included in these studies (e.g., Hilger et al. (2025) and Houde et al. (2019) included more than seven different types of CD, while W. Li et al. (2019) included two different subtypes of spinocerebellar ataxia). In addition, the magnitude of this phonatory compensation can be decreased with cerebellar continuous theta burst stimulation (Q. Lin et al., 2022). Both W. Li et al. (2019) and Q. Lin et al. (2022) also found that IWCD show reduced amplitude of the P2 component in their AEP following *f*_*o*_ perturbations, and this P2 amplitude was enhanced with cerebellar continuous theta burst stimulation (Q. Lin et al., 2022). However, a recent study did not replicate the enhanced phonatory compensation among IWCD despite having tested a variety of perturbation designs (Slis & Parrell, 2026). Instead, in this study, IWCD showed overall similar level of phonatory compensation as their age-matched controls.

#### Articulation

Two studies have been published on articulatory compensation to unexpected formant perturbations in CD (Parrell et al., 2017, 2021). However, these studies produced contradictory results. Parrell et al. (2017) reported increased formant compensation among IWCD versus controls, whereas Parrell et al. (2021) found no significant difference in formant compensation behavior between these groups. Both studies followed generally the same experimental procedure with only minor differences such as using different target words with the /ɛ/ vowel, and a small difference in perturbation magnitude (±150 Hz vs. ±125 mel F1 shift). Thus, the effect of CD on articulatory compensation may be less robust than the effect on phonatory compensation.

### Auditory-Motor Adaptation to Predictable Perturbations in CD

#### Phonation

So far, there is one study that examined auditory-motor adaptation in the phonatory system in IWCD (Slis & Parrell, 2026). Results showed that, unlike age-matched controls, IWCD did not show any phonatory adaptation in either a word production task or a sustained vocalization task.

#### Articulation

Two studies have been published on auditory-motor adaptation to a predictable formant perturbation in IWCD (Parrell et al., 2017, 2021). Both studies reported reduced articulatory adaptation among IWCD versus controls (Parrell et al., 2017, 2021). The studies used the same subject inclusion criteria (different types of cerebellar degeneration were included) and similar formant perturbation (−150 Hz and −125 mel F1 shift, respectively), but Parrell et al. (2017) tested both a gradual and a sudden perturbation schedule whereas Parrell et al. (2021) only tested with the sudden perturbation schedule. IWCD adapted less than controls under both perturbation schedules (Parrell et al., 2017). Instead of such widely used spectral perturbations, a recent study with IWCD examined adaptation in response to temporal perturbations, in particular a predictable and gradual increase in vowel duration (Karlin & Parrell, 2026). Unlike age-matched control participants’ gradual decrease in produced vowel duration, IWCD did not adapt to the perturbation and even showed a slight but significant following response at the group level. The same study also found that IWCD, as a group, showed higher perceptual discrimination thresholds for vowel duration in a separate temporal perception task, but no significant correlation was found between perceptual threshold and adaptation magnitude (Karlin & Parrell, 2026). Overall, to date, studies have consistently found a detrimental effect of CD on adaptation.

### Discussion—Cerebellar Degeneration

Table 6 summarizes the results from all studies on the effects of CD (excluding ET) on speech auditory-motor compensation and adaptation. In sum, current evidence suggests that (1) CD may cause exaggerated phonatory compensation; (2) CD may cause exaggerated articulatory compensation; (3) CD may cause phonatory adaptation to be abolished; (4) CD causes diminished articulatory adaptation.

**Table 6.** Summary of the number of studies on auditory-motor compensation and adaptation among individuals with cerebellar degeneration, separated by reported group effects.

	Compensation	Adaptation
Phonation	• CD > control (3) • CD ≈ control (1)	• CD < control (1)
Articulation	• CD > control (1) • CD ≈ control (1)	• CD < control (3)

*Note*. Same notations as Table 1. CD = cerebellar degeneration.

The exaggerated phonatory compensation in CD resembles exaggerated phonatory compensation in PD. Again, according to computational models of speech sensorimotor control, exaggerated compensation may be explained as an increased sensitivity to auditory feedback, a decreased sensitivity to somatosensory feedback, and a weakened feedforward control system (Weerathunge, Tomassi, et al., 2022). Given that the cerebellum is implicated in both feedback and feedforward control mechanisms, all three hypotheses are possible, and they are not mutually exclusive. A recent computational study that fitted the data from Houde et al. (2019) with a state feedback control model suggested that the difference in phonatory compensation between IWCD and controls can be explained as an overreliance on auditory feedback over somatosensory feedback, and an enhanced controller gain that increases laryngeal motor responses to target errors (Gaines et al., 2024). However, it should be noted that a recent study was unable to replicate the finding of exaggerated phonatory compensation despite having tested multiple variants of the auditory-motor perturbation paradigm (Slis & Parrell, 2026).

Interestingly, although both IWCD and IWPD show exaggerated phonatory compensation, the amplitude of the *f*_*o*_ perturbation-induced P2, an ERP component, is smaller than healthy controls in IWCD (W. Li et al., 2019; Q. Lin et al., 2022), but larger than healthy controls in IWPD (Huang et al., 2016, 2019; Y. Li et al., 2021). This indicates that different neural mechanisms underlie the apparently similar issues with phonatory compensation in PD and CD. It has been suggested that, instead of differences related to sensorimotor integration, the reduced P2 amplitude in CD may indicate that the exaggerated phonatory compensation is caused by impairments in high-level, top-down control of speech production, possibly related to the connections between the cerebellum and cognitive regions such as prefrontal cortex (W. Li et al., 2019; Q. Lin et al., 2022). This idea is in line with known connections between the cerebellum and prefrontal areas (Desmond et al., 1997), and with findings indicating that conditions with prominent prefrontal involvement, such as Alzheimer’s disease, are also associated with exaggerated phonatory compensation (Ranasinghe et al., 2017, 2019). Future studies that combine innovative experimental designs and neuroimaging techniques are needed to disentangle the respective contributions of feedback processing, feedforward movement control, and high-level cognitive control to speech production in these populations.

Adaptation in response to formant, *f*_*o*_, duration and perturbations has consistently been found to be decreased or even abolished in IWCD (Karlin & Parrell, 2026; Parrell et al., 2017, 2021; Slis & Parrell, 2026). In fact, a decrease in sensorimotor adaptation associated with CD has been found across motor domains, including arm movements (Donchin et al., 2012; Hashimoto et al., 2015; Taylor et al., 2010), saccades (Cheviet et al., 2022), and locomotion (Statton et al., 2018). Such reduced adaptation indicates impairments in the internal models that predict sensory consequences of motor commands, and an inefficient updating process of such internal models through sensory prediction errors. Importantly, the cerebellum is considered critical in storing and updating such internal sensorimotor models (Bastian, 2006; Doya, 2000; Krakauer et al., 2019; Miall & Wolpert, 1996). Reduced articulatory adaptation among IWCD is consistent with this perspective. Further computational work such as that of Gaines et al. (2024) may elucidate the underlying mechanistic differences (e.g., feedback sensitivity vs. feedforward gain) that best explain the consistently observed decrease in articulatory adaptation in IWCD.

Similar to PD, studies on CD show that atypical phonatory compensation is more consistent than atypical articulatory compensation. This again may indicate that the phonatory subsystem places more weight on the feedback control mechanism, especially the auditory feedback, whereas the articulatory system may rely more on the feedforward control mechanism (Cowie & Douglas-Cowie, 1992; Kapsner-Smith et al., 2024; Xu et al., 2020).

Even though converging evidence suggests that ET is strongly associated with cerebellar pathologies and may even be considered the most common form of cerebellar degeneration (Louis, 2018; Louis & Faust, 2020a, 2020b; Pan & Kuo, 2022), to date no auditory-motor perturbation studies have included IWET. This is likely due to the fact that most IWET do not exhibit prominent speech symptoms other than essential voice tremor. Similarly, no studies have been conducted on speech-related sensory processing in ET. Based on the consistent effects of other types of CD on phonatory compensation and articulatory adaptation reviewed above, we suggest that investigating auditory-motor compensation and adaptation in ET may offer deeper insights into the role of the cerebellum in the sensorimotor control of speech.

In fact, Vim-DBS in ET offers a unique opportunity to study the role of the cortico-cerebello-thalamo-cortical circuit in speech sensorimotor control. The Vim nucleus of the thalamus receives information from deep cerebellar nuclei, and it is a critical information relay station in this circuit (Milosevic et al., 2018; Papavassiliou et al., 2004). Therefore, studying the effects of Vim-DBS in speech auditory-motor perturbation paradigms may reveal potential causal links between the cerebello-thalamo-cortical circuit and the speech sensorimotor control mechanisms. Notably, outside the speech domain, it was found that Vim-DBS reduced sensorimotor adaptation in arm reaching movements performed in a dynamic force field, and this result was attributed to Vim-DBS disrupting the cerebellar signals necessary for sensorimotor calibration (H. Chen et al., 2006). It remains to be seen whether Vim-DBS has a similar detrimental effect on speech auditory-motor adaptation.

## GENERAL DISCUSSION AND FUTURE DIRECTIONS

This review paper provides an overview of published studies on auditory-motor compensation and auditory-motor adaptation in three groups of basal ganglia and cerebellar disorders: Parkinson’s disease, dystonia (especially laryngeal dystonia), and cerebellar degeneration (including essential tremor). These findings are discussed together with the broader context of speech-related motor and sensory deficits associated with these disorders. The effects of treatment options such as medication and deep brain stimulation are also discussed. Our review was motivated by the fact that the basal ganglia and the cerebellum are both considered critical to feedforward and feedback control mechanisms in computational models of speech sensorimotor control (Parrell & Houde, 2019; Tourville & Guenther, 2011), and auditory-motor compensation and adaptation paradigms allow these control mechanisms to be studied in speech production (Burnett et al., 1998; Houde & Jordan, 1998). Overall, our review revealed that the most consistent findings are exaggerated phonatory compensation in IWPD without dopaminergic medication (X. Chen et al., 2013; Dai et al., 2022; Huang et al., 2016, 2019; Y. Li et al., 2021; Liu et al., 2012; Mollaei et al., 2016), exaggerated phonatory compensation in IWCD (Houde et al., 2019; W. Li et al., 2019; Q. Lin et al., 2022), and diminished articulatory adaptation in IWCD (Parrell et al., 2017, 2021).

The diminished articulatory adaptation in IWCD is consistent with the view that the cerebellum plays a key role in updating feedforward motor commands based on predictive sensory errors (Bastian, 2006; Doya, 2000; Krakauer et al., 2019), which has been confirmed across motor domains (Cheviet et al., 2022; Donchin et al., 2012; Hashimoto et al., 2015; Statton et al., 2018; Taylor et al., 2010). Interestingly, although PD and CD appear to cause similarly exaggerated phonatory compensation, EEG studies show that they are associated with distinct neurophysiological responses to the auditory feedback (Huang et al., 2016, 2019; W. Li et al., 2019; Y. Li et al., 2021; Q. Lin et al., 2022). We suggest that whereas the exaggerated phonatory compensation in PD may relate to changes in sensorimotor brain regions modulated by the cortico-basal ganglia-thalamo-cortical circuit, such as the supplementary motor area (Dai et al., 2022), the exaggerated phonatory compensation in CD may relate to deficits in higher level control of speech production through connections between the cerebellum and prefrontal regions (Q. Lin et al., 2022). More rigorous and hypothesis-driven neuroimaging studies are needed to test these hypotheses.

Our review also revealed many gaps and inconsistencies in the current literature. These include the effects of DBS on phonatory and articulatory adaptation in PD, the effects of LD on phonatory and articulatory adaptation, and the effects of ET and Vim-DBS on any kind of speech compensation or adaptation, among others. Crucially, we suggest that investigating auditory-motor compensation and adaptation in ET, especially the effects of Vim-DBS, will offer particularly valuable insights into the neural mechanisms of speech sensorimotor control. Although ET is strongly associated with cerebellar degeneration (Louis, 2018; Louis & Faust, 2020a, 2020b; Pan & Kuo, 2022), no studies to date have investigated its effect on speech auditory-motor adaptation. Furthermore, Vim-DBS, a common treatment option for medication-refractory ET, offers a unique opportunity to examine the role of the cortico-cerebello-thalamo-cortical circuit in speech sensorimotor control in a direct and causal manner. The Vim nucleus of the thalamus receives information from deep cerebellar nuclei, and it is a critical information relay station in this circuit (Milosevic et al., 2018; Papavassiliou et al., 2004). Studying the acute and longitudinal effects of Vim-DBS on auditory-motor compensation and adaptation can therefore offer a direct, mechanistic understanding of how the cortico-cerebello-thalamo-cortical circuit modulates the speech sensorimotor control system.

An additional issue identified through this review is that, across disorders, results from phonatory adaptation and articulatory compensation studies appear much more inconsistent than those from phonatory compensation and articulatory adaptation studies. In PD, without controlling for medication, one study found smaller phonatory adaptation in IWPD versus controls (Abur et al., 2018), and one study found no group difference (Abur et al., 2021). Similarly, in CD, one study found larger articulatory compensation in IWCD versus controls (Parrell et al., 2017), and one study found no group difference (Parrell et al., 2021). First, it should be noted that the apparent inconsistency may be due to the fact that these two phenomena have not been studied as intensively as either phonatory compensation or articulatory adaptation. However, there is evidence to suggest that the phonatory and articulatory subsystems are fundamentally different with regard to the underlying control mechanisms. When experiencing simultaneous perturbations of *f*_*o*_ and formants, typical speakers show larger phonatory compensation than articulatory compensation (Xu et al., 2020). In typical speakers, test–retest reliability is also higher for phonatory compensation and articulatory adaptation than for phonatory adaptation and articulatory compensation, respectively (Kapsner-Smith et al., 2024). Furthermore, post-lingual deafness causes more rapid degradation of phonation than articulation (Cowie & Douglas-Cowie, 1992). Thus, it can be argued that, on one hand, the phonatory subsystem relies more on feedback control based on online auditory errors and its feedforward system is less prone to updating motor commands based on these errors. On the other hand, the articulatory subsystem is less reliant on online feedback control, at least for auditory errors, and its feedforward control system is more likely to update motor commands based on these errors. These discrepancies also raise the question whether compensation and adaptation are related or independent mechanisms in each subsystem, as recent studies has found no correlation between articulatory compensation and adaptation (Daliri, 2021; Raharjo et al., 2021).

Lastly, although we have categorized the disorders reviewed here into basal ganglia disorders (PD and dystonia) and cerebellar disorders (CD, including ET), it should be noted that the basal ganglia and cerebellar systems are not independent of one another, and that the underlying pathologies of each disorder also cannot be entirely separated just based on these two structures. The basal ganglia and the cerebellum not only influence each other through their respective closed-loop circuits with the cerebral cortex, but also communicate through direct, disynaptic connections, e.g., from the STN to the cerebellar cortex and from the dentate nucleus to the striatum (Bostan et al., 2010; Bostan & Strick, 2018). In fact, IWPD have also been found to show structural and functional impairments associated with the cerebellum (Wu & Hallett, 2013). Therefore, to arrive at a more complete picture of the functions of the basal ganglia and cerebellar networks in the speech sensorimotor control system, it is advisable that future studies on speech compensation and adaptation also include neuroimaging analyses of the specific neural networks affected in individual participants.

In sum, the current review confirmed certain consistent effects of basal ganglia and cerebellar disorders on speech auditory-motor compensation and adaptation, but we also identified many inconsistencies and gaps in our current knowledge. With more rigorous experimental control, richer neuroimaging data, and the incorporation of new techniques such as DBS, we believe that future studies on these disorders can further clarify the roles of the basal ganglia and the cerebellar networks in speech sensorimotor sensorimotor control and offer mechanistic insights as well as clinical management strategies for the motor speech impairments associated with these disorders.

## FUNDING INFORMATION

Hantao Wang, National Science Foundation (https://dx.doi.org/10.13039/100000001), Award ID: 2416385. Hantao Wang, National Institute on Aging (https://dx.doi.org/10.13039/100000049), Award ID: F99AG088438. Ludo Max, National Institute on Deafness and Other Communication Disorders (https://dx.doi.org/10.13039/100000055), Award ID: R01DC020707. Ludo Max, National Institute on Deafness and Other Communication Disorders (https://dx.doi.org/10.13039/100000055), Award ID: R01DC017444. Hantao Wang, National Institute on Aging (https://dx.doi.org/10.13039/100000049), Award ID: K00AG088438. Ludo Max, National Institute on Deafness and Other Communication Disorders (https://dx.doi.org/10.13039/100000055), Award ID: R21DC021557.

## AUTHOR CONTRIBUTIONS

**Hantao Wang**: Conceptualization: Lead; Funding acquisition: Supporting; Writing – original draft: Lead; Writing – review & editing: Equal. **Ludo Max**: Conceptualization: Equal; Funding acquisition: Lead; Supervision: Lead; Writing – review & editing: Equal.

## Notes

^1^ Some researchers suggest that speech changes under predictable auditory perturbations involve both feedback-based compensation and feedforward-based adaptation, and some computational models propose that adaptation may be driven by feedback-based compensation (Kearney et al., 2020). Here, we consider speech changes under predictable auditory perturbations to reflect feedforward adaptation for two reasons. First, feedback-based compensation has a latency of 150–200 ms (Raharjo et al., 2021; Tourville et al., 2008). Vowel durations in single word productions can fall well within that range (Hillenbrand et al., 2001) and then do not provide enough time for compensation. In addition, in most adaptation studies, acoustic measurements are taken from an early or middle portion of the vowel. Given the aforementioned latency, the contribution of feedback mechanisms to formant measurements at those time points will be minimal. Lastly, other computational models do not assume a direct relation between adaptation and compensation (Parrell et al., 2019) and some experimental studies found the two behaviors to be independent (Daliri, 2021; Raharjo et al., 2021).^2^ The fundamental frequency (*f*_*o*_) of the speech signal is associated with the perceived pitch of the human voice and is determined by the vibratory frequency of the vocal folds, which is in turn controlled by activity of the intrinsic laryngeal muscles.^3^ The formants are the resonance frequencies of the vocal tract. For vowel production, the values of the two lowest-frequency formants (F1 and F2) roughly correlate with the degree of oral opening (F1) and anterior-posterior tongue position (F2).

## TECHNICAL TERMS

Feedback control: Modification of ongoing movements based on mismatches between sensory feedback and predicted sensory outcomes of the motor commands.
Feedforward control: Execution of preplanned motor commands to achieve a movement goal and updating future motor commands based on past sensory errors.
Internal models: Neural representations of the mapping between motor commands and their sensory consequences; forward models predict sensory outcomes, inverse models compute required motor commands.
Auditory-motor compensation: An immediate, within-trial corrective response in speech production in response to an unpredictable perturbation of the auditory feedback.
Auditory-motor adaptation: A gradual, trial-by-trial change in speech production in response to a consistent and predictable perturbation of the auditory feedback.

## References

[bib1] Abeyesekera, A., Adams, S., Mancinelli, C., Knowles, T., Gilmore, G., Delrobaei, M., & Jog, M. (2019). Effects of deep brain stimulation of the subthalamic nucleus settings on voice quality, intensity, and prosody in Parkinson’s disease: Preliminary evidence for speech optimization. Canadian Journal of Neurological Sciences, 46(3), 287–294. 10.1017/cjn.2019.16, 30905324

[bib2] Abur, D., Lester-Smith, R. A., Daliri, A., Lupiani, A. A., Guenther, F. H., & Stepp, C. E. (2018). Sensorimotor adaptation of voice fundamental frequency in Parkinson’s disease. PLOS ONE, 13(1), e0191839. 10.1371/journal.pone.0191839, 29373589 PMC5786318

[bib3] Abur, D., & Stepp, C. E. (2020). Acuity to changes in self-generated vocal pitch in Parkinson’s disease. Journal of Speech, Language, and Hearing Research, 63(9), 3208–3214. 10.1044/2020_JSLHR-20-00003, 32853119 PMC7890224

[bib4] Abur, D., Subaciute, A., Daliri, A., Lester-Smith, R. A., Lupiani, A. A., Cilento, D., Enos, N. M., Weerathunge, H. R., Tardif, M. C., & Stepp, C. E. (2021). Feedback and feedforward auditory-motor processes for voice and articulation in Parkinson’s disease. Journal of Speech, Language, and Hearing Research, 64(12), 4682–4694. 10.1044/2021_JSLHR-21-00153, 34731577 PMC9150666

[bib5] Ackermann, H., Gräber, S., Hertrich, I., & Daum, I. (1997). Categorical speech perception in cerebellar disorders. Brain and Language, 60(2), 323–331. 10.1006/brln.1997.1826, 9344481

[bib6] Ackermann, H., Gräber, S., Hertrich, I., & Daum, I. (1999a). Cerebellar contributions to the perception of temporal cues within the speech and nonspeech domain. Brain and Language, 67(3), 228–241. 10.1006/brln.1999.2056, 10210632

[bib7] Ackermann, H., Gräber, S., Hertrich, I., & Daum, I. (1999b). Phonemic vowel length contrasts in cerebellar disorders. Brain and Language, 67(2), 95–109. 10.1006/brln.1998.2044, 10092344

[bib9] Ackermann, H., & Hertrich, I. (1997). Voice onset time in ataxic dysarthria. Brain and Language, 56(3), 321–333. 10.1006/brln.1997.1740, 9070415

[bib8] Ackermann, H., Hertrich, I., & Hehr, T. (1995). Oral diadochokinesis in neurological dysarthrias. Folia Phoniatrica et Logopaedica, 47(1), 15–23. 10.1159/000266338, 7728177

[bib10] Ackermann, H., & Ziegler, W. (1991). Articulatory deficits in parkinsonian dysarthria: An acoustic analysis. Journal of Neurology, Neurosurgery, and Psychiatry, 54(12), 1093–1098. 10.1136/jnnp.54.12.1093, 1783924 PMC1014687

[bib11] Ackermann, H., & Ziegler, W. (1994). Acoustic analysis of vocal instability in cerebellar dysfunctions. Annals of Otology, Rhinology, and Laryngology, 103(2), 98–104. 10.1177/000348949410300203, 8311397

[bib12] Airaksinen, K., Mäkelä, J. P., Taulu, S., Ahonen, A., Nurminen, J., Schnitzler, A., & Pekkonen, E. (2011). Effects of DBS on auditory and somatosensory processing in Parkinson’s disease. Human Brain Mapping, 32(7), 1091–1099. 10.1002/hbm.21096, 20645306 PMC6870287

[bib13] Akkunje, P. S., Yamini, B. K., Yadav, R., Shivashankar, N., Malo, P. K., Thennarasu, K., Hegde, S., & Pal, P. K. (2021). Phonatory characteristics of male patients with classic essential tremor. Journal of Movement Disorders, 14(3), 226–230. 10.14802/jmd.21010, 34399566 PMC10657739

[bib14] Aldridge, D., Theodoros, D., Angwin, A., & Vogel, A. P. (2016). Speech outcomes in Parkinson’s disease after subthalamic nucleus deep brain stimulation: A systematic review. Parkinsonism & Related Disorders, 33, 3–11. 10.1016/j.parkreldis.2016.09.022, 27693195

[bib15] Alomar, S., King, N. K. K., Tam, J., Bari, A. A., Hamani, C., & Lozano, A. M. (2017). Speech and language adverse effects after thalamotomy and deep brain stimulation in patients with movement disorders: A meta-analysis. Movement Disorders, 32(1), 53–63. 10.1002/mds.26924, 28124434

[bib16] Amantini, A., Rossi, L., De Scisciolo, G., Bindi, A., Pagnini, P., & Zappoli, R. (1984). Auditory evoked potentials (early, middle, late components) and audiological tests in Friedreich’s ataxia. Electroencephalography and Clinical Neurophysiology, 58(1), 37–47. 10.1016/0013-4694(84)90198-6, 6203701

[bib17] Artieda, J., Pastor, M. A., Lacruz, F., & Obeso, J. A. (1992). Temporal discrimination is abnormal in Parkinson’s disease. Brain, 115(1), 199–210. 10.1093/brain/115.1.199, 1559154

[bib18] Ashkan, K., Rogers, P., Bergman, H., & Ughratdar, I. (2017). Insights into the mechanisms of deep brain stimulation. Nature Reviews Neurology, 13(9), 548–554. 10.1038/nrneurol.2017.105, 28752857

[bib19] Avecillas-Chasin, J. M., Poologaindran, A., Morrison, M. D., Rammage, L. A., & Honey, C. R. (2018). Unilateral thalamic deep brain stimulation for voice tremor. Stereotactic and Functional Neurosurgery, 96(6), 392–399. 10.1159/000495413, 30625492

[bib20] Bahners, B. H., Florin, E., Rohrhuber, J., Krause, H., Hirschmann, J., van de Vijver, R., Schnitzler, A., & Butz, M. (2020). Deep brain stimulation does not modulate auditory-motor integration of speech in Parkinson’s disease. Frontiers in Neurology, 11, 655. 10.3389/fneur.2020.00655, 32754112 PMC7366847

[bib21] Baker, K. K., Ramig, L. O., Luschei, E. S., & Smith, M. E. (1998). Thyroarytenoid muscle activity associated with hypophonia in Parkinson disease and aging. Neurology, 51(6), 1592–1598. 10.1212/WNL.51.6.1592, 9855507

[bib22] Balint, B., Mencacci, N. E., Valente, E. M., Pisani, A., Rothwell, J., Jankovic, J., Vidailhet, M., & Bhatia, K. P. (2018). Dystonia. Nature Reviews Disease Primers, 4(1), 25. 10.1038/s41572-018-0023-6, 30237473

[bib23] Bandini, A., Orlandi, S., Giovannelli, F., Felici, A., Cincotta, M., Clemente, D., Vanni, P., Zaccara, G., & Manfredi, C. (2016). Markerless analysis of articulatory movements in patients with Parkinson’s disease. Journal of Voice, 30(6), 766.e1–766.e11. 10.1016/j.jvoice.2015.10.014, 26620259

[bib24] Barbe, M. T., Dembek, T. A., Becker, J., Raethjen, J., Hartinger, M., Meister, I. G., Runge, M., Maarouf, M., Fink, G. R., & Timmermann, L. (2014). Individualized current-shaping reduces DBS-induced dysarthria in patients with essential tremor. Neurology, 82(7), 614–619. 10.1212/WNL.0000000000000127, 24443448 PMC3963416

[bib25] Bastian, A. J. (2006). Learning to predict the future: The cerebellum adapts feedforward movement control. Current Opinion in Neurobiology, 16(6), 645–649. 10.1016/j.conb.2006.08.016, 17071073

[bib26] Becker, J., Barbe, M. T., Hartinger, M., Dembek, T. A., Pochmann, J., Wirths, J., Allert, N., Mücke, D., Hermes, A., Meister, I. G., Visser-Vandewalle, V., Grice, M., & Timmermann, L. (2017). The effect of uni- and bilateral thalamic deep brain stimulation on speech in patients with essential tremor: Acoustics and intelligibility. Neuromodulation, 20(3), 223–232. 10.1111/ner.12546, 28160355

[bib27] Becker, J., Thies, T., Petry-Schmelzer, J. N., Dembek, T. A., Reker, P., Mücke, D., Grice, M., Visser-Vandewalle, V., Fink, G. R., & Barbe, M. T. (2020). The effects of thalamic and posterior subthalamic deep brain stimulation on speech in patients with essential tremor—A prospective, randomized, doubleblind crossover study. Brain and Language, 202, 104724. 10.1016/j.bandl.2019.104724, 31884313

[bib28] Behroozmand, R., Johari, K., Kelley, R. M., Kapnoula, E. C., Narayanan, N. S., & Greenlee, J. D. W. (2019). Effect of deep brain stimulation on vocal motor control mechanisms in Parkinson’s disease. Parkinsonism & Related Disorders, 63, 46–53. 10.1016/j.parkreldis.2019.03.002, 30871801 PMC9585766

[bib29] Bhatia, K. P., & Marsden, C. D. (1994). The behavioural and motor consequences of focal lesions of the basal ganglia in man. Brain, 117(4), 859–876. 10.1093/brain/117.4.859, 7922471

[bib30] Bodranghien, F., Bastian, A., Casali, C., Hallett, M., Louis, E. D., Manto, M., Mariën, P., Nowak, D. A., Schmahmann, J. D., Serrao, M., Steiner, K. M., Strupp, M., Tilikete, C., Timmann, D., & van Dun, K. (2016). Consensus paper: Revisiting the symptoms and signs of cerebellar syndrome. Cerebellum, 15(3), 369–391. 10.1007/S12311-015-0687-3, 26105056 PMC5565264

[bib31] Bostan, A. C., Dum, R. P., & Strick, P. L. (2010). The basal ganglia communicate with the cerebellum. Proceedings of the National Academy of Sciences of the United States of America, 107(18), 8452–8456. 10.1073/pnas.1000496107, 20404184 PMC2889518

[bib32] Bostan, A. C., & Strick, P. L. (2018). The basal ganglia and the cerebellum: Nodes in an integrated network. Nature Reviews Neuroscience, 19(6), 338–350. 10.1038/S41583-018-0002-7, 29643480 PMC6503669

[bib33] Boutsen, F., Duffy, J. R., Dimassi, H., & Christman, S. S. (2011). Long-term phonatory instability in ataxic dysarthria. Folia Phoniatrica et Logopaedica, 63(4), 216–220. 10.1159/000319971, 21088427

[bib34] Bradshaw, A. R., Lametti, D. R., & McGettigan, C. (2021). The role of sensory feedback in developmental stuttering: A review. Neurobiology of Language, 2(2), 308–334. 10.1162/nol_a_00036, 37216145 PMC10158644

[bib35] Brajot, F.-X., & Neiman, A. B. (2020). Vocal wow in an adapted reflex resonance model. The Journal of the Acoustical Society of America, 147(3), 1822–1833. 10.1121/10.0000938, 32237861

[bib36] Brajot, F.-X., Shiller, D. M., & Gracco, V. L. (2016). Autophonic loudness perception in Parkinson’s disease. The Journal of the Acoustical Society of America, 139(3), 1364–1371. 10.1121/1.4944569, 27036273 PMC4818272

[bib37] Brendel, B., Lowit, A., & Howell, P. (2004). The effects of delayed and frequency shifted feedback on speakers with Parkinson’s disease. Journal of Medical Speech-Language Pathology, 12, 131–138. 19330038 PMC2661057

[bib38] Brittain, J.-S., & Brown, P. (2014). Oscillations and the basal ganglia: Motor control and beyond. NeuroImage, 85(Part 2), 637–647. 10.1016/j.neuroimage.2013.05.084, 23711535 PMC4813758

[bib39] Burnett, T. A., Freedland, M. B., Larson, C. R., & Hain, T. C. (1998). Voice F0 responses to manipulations in pitch feedback. The Journal of the Acoustical Society of America, 103(6), 3153–3161. 10.1121/1.423073, 9637026

[bib40] Caligiuri, M. P., & Abbs, J. H. (1987). Response properties of the perioral reflex in Parkinson’s disease. Experimental Neurology, 98(3), 563–572. 10.1016/0014-4886(87)90265-2, 3678433

[bib41] Cameron, D. J., Pickett, K. A., Earhart, G. M., & Grahn, J. A. (2016). The effect of dopaminergic medication on beat-based auditory timing in Parkinson’s disease. Frontiers in Neurology, 7, 19. 10.3389/fneur.2016.00019, 26941707 PMC4761840

[bib42] Cannito, M. P., Chorna, L. B., Kahane, J. C., & Dworkin, J. P. (2014). Influence of consonant voicing characteristics on sentence production in abductor versus adductor spasmodic dysphonia. Journal of Voice, 28(3), 394.e13–394.e22. 10.1016/j.jvoice.2013.10.010, 24440058

[bib43] Carpenter, M. A., Pahwa, R., Miyawaki, K. L., Wilkinson, S. B., Searl, J. P., & Koller, W. C. (1998). Reduction in voice tremor under thalamic stimulation. Neurology, 50(3), 796–798. 10.1212/WNL.50.3.796, 9521280

[bib44] Cassandro, E., Mosca, F., Sequino, L., De Falco, F. A., & Campanella, G. (1986). Otoneurological findings in Friedreich’s ataxia and other inherited neuropathies. Audiology, 25(2), 84–91. 10.3109/00206098609078373, 3707440

[bib45] Chen, H., Hua, S. E., Smith, M. A., Lenz, F. A., & Shadmehr, R. (2006). Effects of human cerebellar thalamus disruption on adaptive control of reaching. Cerebral Cortex, 16(10), 1462–1473. 10.1093/cercor/bhj087, 16357337 PMC1560096

[bib46] Chen, X., Zhu, X., Wang, E. Q., Chen, L., Li, W., Chen, Z., & Liu, H. (2013). Sensorimotor control of vocal pitch production in Parkinson’s disease. Brain Research, 1527, 99–107. 10.1016/j.brainres.2013.06.030, 23820424

[bib47] Chen, Y.-W., & Watson, P. J. (2017). Speech production and sensory impairment in mild Parkinson’s disease. The Journal of the Acoustical Society of America, 141(5), 3030–3041. 10.1121/1.4980138, 28599574

[bib48] Chen, Z., Li, J., Ren, Q., & Ge, P. (2019). Acoustic and perceptual analyses of adductor spasmodic dysphonia in Mandarin-speaking Chinese. Journal of Voice, 33(3), 333–339. 10.1016/j.jvoice.2017.12.007, 29449062

[bib49] Cheviet, A., Masselink, J., Koun, E., Salemme, R., Lappe, M., Froment-Tilikete, C., & Pélisson, D. (2022). Cerebellar signals drive motor adjustments and visual perceptual changes during forward and backward adaptation of reactive saccades. Cerebral Cortex, 32(18), 3896–3916. 10.1093/cercor/bhab455, 34979550

[bib50] Cimino-Knight, A. M., & Sapienza, C. M. (2001). Consistency of voice produced by patients with adductor spasmodic dysphonia: A preliminary investigation. Journal of Speech, Language, and Hearing Research, 44(4), 793–802. 10.1044/1092-4388(2001/062), 11521772

[bib51] Clark, J. P., Adams, S. G., Dykstra, A. D., Moodie, S., & Jog, M. (2014). Loudness perception and speech intensity control in Parkinson’s disease. Journal of Communication Disorders, 51, 1–12. 10.1016/j.jcomdis.2014.08.001, 25194745

[bib52] Conte, A., Ferrazzano, G., Manzo, N., Leodori, G., Fabbrini, G., Fasano, A., Tinazzi, M., & Berardelli, A. (2015). Somatosensory temporal discrimination in essential tremor and isolated head and voice tremors. Movement Disorders, 30(6), 822–827. 10.1002/mds.26163, 25736856

[bib53] Conte, A., Khan, N., Defazio, G., Rothwell, J. C., & Berardelli, A. (2013). Pathophysiology of somatosensory abnormalities in Parkinson disease. Nature Reviews Neurology, 9(12), 687–697. 10.1038/nrneurol.2013.224, 24217516

[bib54] Coutinho, S. B., Diaféria, G., Oliveira, G., & Behlau, M. (2009). Voice and speech of individuals with Parkinson’s disease during amplification, delay and masking situations. Pro-Fono, 21(3), 219–224. 10.1590/S0104-56872009000300007, 19838568

[bib55] Cowie, R., & Douglas-Cowie, E. (1992). Postlingually acquired deafness: Speech deterioration and the wider consequences. Mouton de Gruyter. 10.1515/9783110869125

[bib56] Cushnie-Sparrow, D., Adams, S., Abeyesekera, A., Pieterman, M., Gilmore, G., & Jog, M. (2018). Voice quality severity and responsiveness to levodopa in Parkinson’s disease. Journal of Communication Disorders, 76, 1–10. 10.1016/j.jcomdis.2018.07.003, 30053648

[bib57] Dai, G., Wang, M., Li, Y., Guo, Z., Jones, J. A., Li, T., Chang, Y., Wang, E. Q., Chen, L., Liu, P., Chen, X., & Liu, H. (2022). Continuous theta burst stimulation over left supplementary motor area facilitates auditory-vocal integration in individuals with Parkinson’s disease. Frontiers in Aging Neuroscience, 14, 948696. 10.3389/fnagi.2022.948696, 36051304 PMC9426458

[bib58] Daliri, A. (2021). A computational model for estimating the speech motor system’s sensitivity to auditory prediction errors. Journal of Speech, Language, and Hearing Research, 64(6), 1841–1854. 10.1044/2021_JSLHR-20-00484, 34043445 PMC8740760

[bib59] Daliri, A., Heller Murray, E. S., Blood, A. J., Burns, J., Noordzij, J. P., Nieto-Castanon, A., Tourville, J. A., & Guenther, F. H. (2020). Auditory feedback control mechanisms do not contribute to cortical hyperactivity within the voice production network in adductor spasmodic dysphonia. Journal of Speech, Language, and Hearing Research, 63(2), 421–432. 10.1044/2019_JSLHR-19-00325, 32091959 PMC7210444

[bib60] Darley, F. L., Aronson, A. E., & Brown, J. R. (1969). Differential diagnostic patterns of dysarthria. Journal of Speech and Hearing Research, 12(2), 246–269. 10.1044/jshr.1202.246, 5808852

[bib61] Darling, M., & Huber, J. E. (2011). Changes to articulatory kinematics in response to loudness cues in individuals with Parkinson’s disease. Journal of Speech, Language, and Hearing Research, 54(5), 1247–1259. 10.1044/1092-4388(2011/10-0024), 21386044 PMC3433496

[bib62] De Keyser, K., De Letter, M., Santens, P., Talsma, D., Botteldooren, D., & Bockstael, A. (2021). Neurophysiological investigation of auditory intensity dependence in patients with Parkinson’s disease. Journal of Neural Transmission, 128(3), 345–356. 10.1007/S00702-021-02305-3, 33515333

[bib63] De Keyser, K., Santens, P., Bockstael, A., Botteldooren, D., Talsma, D., De Vos, S., Van Cauwenberghe, M., Verheugen, F., Corthals, P., & De Letter, M. (2016). The relationship between speech production and speech perception deficits in Parkinson’s disease. Journal of Speech, Language, and Hearing Research, 59(5), 915–931. 10.1044/2016_JSLHR-S-15-0197, 27617884

[bib64] Deleyiannis, F. W.-B., Gillespie, M., Yamashita, T., Bielamowicz, S., & Ludlow, C. L. (1999). Laryngeal long latency response conditioning in abductor spasmodic dysphonia. Annals of Otology, Rhinology, and Laryngology, 108(6), 612–619. 10.1177/000348949910800615, 10378532

[bib65] Desmond, J. E., Gabrieli, J. D. E., Wagner, A. D., Ginier, B. L., & Glover, G. H. (1997). Lobular patterns of cerebellar activation in verbal working-memory and finger-tapping tasks as revealed by functional MRI. Journal of Neuroscience, 17(24), 9675–9685. 10.1523/JNEUROSCI.17-24-09675.1997, 9391022 PMC6573411

[bib66] Deuschl, G., Raethjen, J., Hellriegel, H., & Elble, R. (2011). Treatment of patients with essential tremor. Lancet Neurology, 10(2), 148–161. 10.1016/S1474-4422(10)70322-7, 21256454

[bib67] Donchin, O., Rabe, K., Diedrichsen, J., Lally, N., Schoch, B., Gizewski, E. R., & Timmann, D. (2012). Cerebellar regions involved in adaptation to force field and visuomotor perturbation. Journal of Neurophysiology, 107(1), 134–147. 10.1152/jn.00007.2011, 21975446

[bib68] Doya, K. (2000). Complementary roles of basal ganglia and cerebellum in learning and motor control. Current Opinion in Neurobiology, 10(6), 732–739. 10.1016/S0959-4388(00)00153-7, 11240282

[bib69] Dromey, C., & Adams, S. (2000). Loudness perception and hypophonia in Parkinson disease. Journal of Medical Speech-Language Pathology, 8(4), 255–259.

[bib70] Dromey, C., Warrick, P., & Irish, J. (2002). The influence of pitch and loudness changes on the acoustics of vocal tremor. Journal of Speech, Language, and Hearing Research, 45(5), 879–890. 10.1044/1092-4388(2002/071), 12381046

[bib71] Duffy, J. (2013). Motor speech disorders: Substrates, differential diagnosis, and management (3rd ed.). Elsevier Mosby.

[bib72] Edgar, J. D., Sapienza, C. M., Bidus, K., & Ludlow, C. L. (2001). Acoustic measures of symptoms in abductor spasmodic dysphonia. Journal of Voice, 15(3), 362–372. 10.1016/S0892-1997(01)00038-8, 11575633

[bib73] Eisinger, R. S., Wong, J., Almeida, L., Ramirez-Zamora, A., Cagle, J. N., Giugni, J. C., Ahmed, B., Bona, A. R., Monari, E., Wagle Shukla, A., Hess, C. W., Hilliard, J. D., Foote, K. D., Gunduz, A., Okun, M. S., & Martinez-Ramirez, D. (2018). Ventral intermediate nucleus versus zona incerta region deep brain stimulation in essential tremor. Movement Disorders Clinical Practice, 5(1), 75–82. 10.1002/mdc3.12565, 30363386 PMC6174382

[bib74] Erickson-DiRenzo, E., Kuijper, F. M., Barbosa, D. A. N., Lim, E. A., Lin, P. T., Lising, M. A., Huang, Y., Sung, C. K., & Halpern, C. H. (2020). Multiparametric laryngeal assessment of the effect of thalamic deep brain stimulation on essential vocal tremor. Parkinsonism & Related Disorders, 81, 106–112. 10.1016/j.parkreldis.2020.10.026, 33120071

[bib75] Evidente, V. G. H., Ponce, F. A., Evidente, M. H., Lambert, M., Garrett, R., Sugumaran, M., & Lott, D. G. (2020). Adductor spasmodic dysphonia improves with bilateral thalamic deep brain stimulation: Report of 3 cases done asleep and review of literature. Tremor and Other Hyperkinetic Movements, 10, 60. 10.5334/tohm.575, 33505767 PMC7792454

[bib76] Fabbri, M., Zibetti, M., Ferrero, G., Accornero, A., Guimaraes, I., Rizzone, M. G., Romagnolo, A., Ferreira, J. J., & Lopiano, L. (2019). Is lowering stimulation frequency a feasible option for subthalamic deep brain stimulation in Parkinson’s disease patients with dysarthria? Parkinsonism & Related Disorders, 64, 242–248. 10.1016/j.parkreldis.2019.04.018, 31060986

[bib77] Fenoy, A. J., McHenry, M. A., & Schiess, M. C. (2017). Speech changes induced by deep brain stimulation of the subthalamic nucleus in Parkinson disease: Involvement of the dentatorubrothalamic tract. Journal of Neurosurgery, 126(6), 2017–2027. 10.3171/2016.5.JNS16243, 27611200

[bib78] Finitzo-Hieber, T., Freeman, F. J., Gerling, I. J., Dobson, L., & Schaefer, S. D. (1982). Auditory brainstem response abnormalities in adductor spasmodic dysphonia. American Journal of Otolaryngology, 3(1), 26–30. 10.1016/S0196-0709(82)80029-X, 7114388

[bib79] Forrest, K., & Weismer, G. (1995). Dynamic aspects of lower lip movement in parkinsonian and neurologically normal geriatric speakers’ production of stress. Journal of Speech and Hearing Research, 38(2), 260–272. 10.1044/jshr.3802.260, 7596092

[bib80] Forrest, K., Weismer, G., & Turner, G. S. (1989). Kinematic, acoustic, and perceptual analyses of connected speech produced by parkinsonian and normal geriatric adults. The Journal of the Acoustical Society of America, 85(6), 2608–2622. 10.1121/1.397755, 2745883

[bib81] Gaines, J. L., Kim, K. S., Parrell, B., Ramanarayanan, V., Pongos, A. L., Nagarajan, S. S., & Houde, J. F. (2024). Bayesian inference of state feedback control parameters for fo perturbation responses in cerebellar ataxia. PLoS Computational Biology, 20(10), e1011986. 10.1371/journal.pcbi.1011986, 39392859 PMC11498721

[bib82] Gallena, S., Smith, P. J., Zeffiro, T., & Ludlow, C. L. (2001). Effects of levodopa on laryngeal muscle activity for voice onset and offset in Parkinson disease. Journal of Speech, Language, and Hearing Research, 44(6), 1284–1299. 10.1044/1092-4388(2001/100), 11776365

[bib83] Gamboa, J., Jiménez-Jiménez, F. J., Nieto, A., Cobeta, I., Vegas, A., Ortí-Pareja, M., Gasalla, T., Molina, J. A., & García-Albea, E. (1998). Acoustic voice analysis in patients with essential tremor. Journal of Voice, 12(4), 444–452. 10.1016/S0892-1997(98)80053-2, 9988031

[bib84] Gamboa, J., Jiménez-Jiménez, F. J., Nieto, A., Montojo, J., Ortí-Pareja, M., Molina, J. A., García-Albea, E., & Cobeta, I. (1997). Acoustic voice analysis in patients with Parkinson’s disease treated with dopaminergic drugs. Journal of Voice, 11(3), 314–320. 10.1016/S0892-1997(97)80010-0, 9297676

[bib85] Gandhi, K. R., & Saadabadi, A. (2023). Levodopa (L-dopa). In Encyclopedia of psychopharmacology (pp. 898–899). Springer. 10.1007/978-3-642-36172-2_200303

[bib86] Gentil, M. (1990). Acoustic characteristics of speech in Friedreich’s disease. Folia Phoniatrica et Logopaedica, 42(3), 125–134. 10.1159/000266056, 2379900

[bib87] Gillivan-Murphy, P., & Miller, N. (2011). Voice tremor: What we know and what we do not know. Current Opinion in Otolaryngology & Head and Neck Surgery, 19(3), 155–159. 10.1097/MOO.0B013E328345970C, 21460728

[bib88] Goberman, A. M., & Elmer, L. W. (2005). Acoustic analysis of clear versus conversational speech in individuals with Parkinson disease. Journal of Communication Disorders, 38(3), 215–230. 10.1016/j.jcomdis.2004.10.001, 15748725

[bib89] Groppa, S., Herzog, J., Falk, D., Riedel, C., Deuschl, G., & Volkmann, J. (2014). Physiological and anatomical decomposition of subthalamic neurostimulation effects in essential tremor. Brain, 137(1), 109–121. 10.1093/brain/awt304, 24277721

[bib90] Grover, T., Georgiev, D., Kalliola, R., Mahlknecht, P., Zacharia, A., Candelario, J., Hyam, J., Zrinzo, L., Hariz, M., Foltynie, T., Limousin, P., Jahanshahi, M., & Tripoliti, E. (2019). Effect of low versus high frequency subthalamic deep brain stimulation on speech intelligibility and verbal fluency in Parkinson’s disease: A double-blind study. Journal of Parkinson’s Disease, 9(1), 141–151. 10.3233/JPD-181368, 30594934

[bib91] Grube, M., Cooper, F. E., Chinnery, P. F., & Griffiths, T. D. (2010). Dissociation of duration-based and beat-based auditory timing in cerebellar degeneration. Proceedings of the National Academy of Sciences of the United States of America, 107(25), 11597–11601. 10.1073/pnas.0910473107, 20534501 PMC2895141

[bib92] Gulberti, A., Hamel, W., Buhmann, C., Boelmans, K., Zittel, S., Gerloff, C., Westphal, M., Engel, A. K., Schneider, T. R., & Moll, C. K. E. (2015). Subthalamic deep brain stimulation improves auditory sensory gating deficit in Parkinson’s disease. Clinical Neurophysiology, 126(3), 565–574. 10.1016/j.clinph.2014.06.046, 25085452

[bib93] Hadjiosif, A. M., Krakauer, J. W., & Haith, A. M. (2021). Did we get sensorimotor adaptation wrong? Implicit adaptation as direct policy updating rather than forward-model-based learning. Journal of Neuroscience, 41(12), 2747–2761. 10.1523/JNEUROSCI.2125-20.2021, 33558432 PMC8018745

[bib94] Hägglund, P., Sandström, L., Blomstedt, P., & Karlsson, F. (2016). Voice tremor in patients with essential tremor: Effects of deep brain stimulation of caudal zona incerta. Journal of Voice, 30(2), 228–233. 10.1016/j.jvoice.2015.04.003, 26001501

[bib95] Haith, A. M., & Krakauer, J. W. (2013). Model-based and model-free mechanisms of human motor learning. Advances in Experimental Medicine and Biology, 782, 1–21. 10.1007/978-1-4614-5465-6_1, 23296478 PMC3570165

[bib96] Hammer, M. J., & Barlow, S. M. (2010). Laryngeal somatosensory deficits in Parkinson’s disease: Implications for speech respiratory and phonatory control. Experimental Brain Research, 201(3), 401–409. 10.1007/S00221-009-2048-2, 20012947 PMC2834233

[bib97] Hanson, D. G., Gerratt, B. R., & Ward, P. H. (1984). Cinegraphic observations of laryngeal function in Parkinson’s disease. Laryngoscope, 94(3), 348–353. 10.1288/00005537-198403000-00011, 6700351

[bib98] Harel, B., Cannizzaro, M., & Snyder, P. J. (2004). Variability in fundamental frequency during speech in prodromal and incipient Parkinson’s disease: A longitudinal case study. Brain and Cognition, 56(1), 24–29. 10.1016/j.bandc.2004.05.002, 15380872

[bib99] Hashimoto, Y., Honda, T., Matsumura, K., Nakao, M., Soga, K., Katano, K., Yokota, T., Mizusawa, H., Nagao, S., & Ishikawa, K. (2015). Quantitative evaluation of human cerebellum-dependent motor learning through prism adaptation of hand-reaching movement. PLOS ONE, 10(3), e0119376. 10.1371/journal.pone.0119376, 25785588 PMC4364988

[bib100] Haslinger, B., Erhard, P., Dresel, C., Castrop, F., Roettinger, M., & Ceballos-Baumann, A. O. (2005). “Silent event-related” fMRI reveals reduced sensorimotor activation in laryngeal dystonia. Neurology, 65(10), 1562–1569. 10.1212/01.WNL.0000184478.59063.DB, 16301482

[bib101] Heinks-Maldonado, T. H., Mathalon, D. H., Gray, M., & Ford, J. M. (2005). Fine-tuning of auditory cortex during speech production. Psychophysiology, 42(2), 180–190. 10.1111/J.1469-8986.2005.00272.X, 15787855

[bib102] Hermes, A., Mücke, D., Thies, T., Barbe, M. T., Hermes, A., Mücke, D., Thies, T., & Barbe, M. T. (2019). Coordination patterns in essential tremor patients with deep brain stimulation: Syllables with low and high complexity. Laboratory Phonology, 10(1), 6. 10.5334/labphon.141

[bib103] Hilger, A., Cole, J., & Larson, C. (2025). Feedback and feedforward control of intonation in cerebellar ataxia. Language, Cognition and Neuroscience, 40(10), 1350–1369. 10.1080/23273798.2025.2541696

[bib104] Hillenbrand, J. M., Clark, M. J., & Nearey, T. M. (2001). Effects of consonant environment on vowel formant patterns. The Journal of the Acoustical Society of America, 109(2), 748–763. 10.1121/1.1337959, 11248979

[bib105] Hirose, H. (1986). Pathophysiology of motor speech disorders (dysarthria). Folia Phoniatrica et Logopaedica, 38(2–4), 61–88. 10.1159/000265824, 3527898

[bib106] Hirose, H., Kiritani, S., Ushijima, T., & Sawashima, M. (1978). Analysis of abnormal articulatory dynamics in two dysarthric patients. Journal of Speech and Hearing Disorders, 43(1), 96–105. 10.1044/jshd.4301.96, 633876

[bib107] Hlavnička, J., Tykalová, T., Ulmanová, O., Dušek, P., Horáková, D., Růžička, E., Klempíř, J., & Rusz, J. (2020). Characterizing vocal tremor in progressive neurological diseases via automated acoustic analyses. Clinical Neurophysiology, 131(5), 1155–1165. 10.1016/j.clinph.2020.02.005, 32146096

[bib108] Ho, A. K., Bradshaw, J. L., & Iansek, R. (2000). Volume perception in parkinsonian speech. Movement Disorders, 15(6), 1125–1131. 10.1002/1531-8257(200011)15:6<1125::aid-mds1010>3.0.co;2-r, 11104195

[bib109] Ho, A. K., Bradshaw, J. L., Iansek, R., & Alfredson, R. (1999). Speech volume regulation in Parkinson’s disease: Effects of implicit cues and explicit instructions. Neuropsychologia, 37(13), 1453–1460. 10.1016/S0028-3932(99)00067-6, 10617265

[bib110] Ho, A. K., Iansek, R., Marigliani, C., Bradshaw, J. L., & Gates, S. (1998). Speech impairment in a large sample of patients with Parkinson’s disease. Behavioural Neurology, 11(3), 131–137. 22387592

[bib111] Holmes, R. J., Oates, J. M., Phyland, D. J., & Hughes, A. J. (2000). Voice characteristics in the progression of Parkinson’s disease. International Journal of Language & Communication Disorders, 35(3), 407–418. 10.1080/136828200410654, 10963022

[bib112] Honey, C. R., Krüger, M. T., Almeida, T., Rammage, L. A., Tamber, M. S., Morrison, M. D., Poologaindran, A., & Hu, A. (2021). Thalamic deep brain stimulation for spasmodic dysphonia: A phase I prospective randomized double-blind crossover trial. Neurosurgery, 89(1), 45–52. 10.1093/neuros/nyab095, 33862624 PMC8223243

[bib113] Hornykiewicz, O. (2002). Dopamine miracle: From brain homogenate to dopamine replacement. Movement Disorders, 17(3), 501–508. 10.1002/mds.10115, 12112197

[bib114] Houde, J. F., Gill, J. S., Agnew, Z., Kothare, H., Hickok, G., Parrell, B., Ivry, R. B., & Nagarajan, S. S. (2019). Abnormally increased vocal responses to pitch feedback perturbations in patients with cerebellar degeneration. The Journal of the Acoustical Society of America, 145(5), EL372–EL378. 10.1121/1.5100910, 31153297 PMC6517184

[bib115] Houde, J. F., & Jordan, M. I. (1998). Sensorimotor adaptation in speech production. Science, 279(5354), 1213–1216. 10.1126/science.279.5354.1213, 9469813

[bib116] Houde, J. F., Nagarajan, S. S., Sekihara, K., & Merzenich, M. M. (2002). Modulation of the auditory cortex during speech: An MEG study. Journal of Cognitive Neuroscience, 14(8), 1125–1138. 10.1162/089892902760807140, 12495520

[bib117] Huang, X., Chen, X., Yan, N., Jones, J. A., Wang, E. Q., Chen, L., Guo, Z., Li, W., Liu, P., & Liu, H. (2016). The impact of Parkinson’s disease on the cortical mechanisms that support auditory-motor integration for voice control. Human Brain Mapping, 37(12), 4248–4261. 10.1002/hbm.23306, 27400999 PMC6867337

[bib118] Huang, X., Fan, H., Li, J., Jones, J. A., Wang, E. Q., Chen, L., Chen, X., & Liu, H. (2019). External cueing facilitates auditory-motor integration for speech control in individuals with Parkinson’s disease. Neurobiology of Aging, 76, 96–105. 10.1016/j.neurobiolaging.2018.12.020, 30710834

[bib119] Hughes, J. T., Brownell, B., & Hewer, R. L. (1968). The peripheral sensory pathway in Friedreich’s ataxia: An examination by light and electron microscopy of the posterior nerve roots, posterior root ganglia, and peripheral sensory nerves in cases of Friedreich’s ataxia. Brain, 91(4), 803–818. 10.1093/brain/91.4.803, 4178703

[bib120] Hunker, C. J., Abbs, J. H., & Barlow, S. M. (1982). The relationship between parkinsonian rigidity and hypokinesia in the orofacial system: A quantitative analysis. Neurology, 32(7), 749–754. 10.1212/WNL.32.7.749, 7201112

[bib121] Jannetts, S., & Lowit, A. (2014). Cepstral analysis of hypokinetic and ataxic voices: Correlations with perceptual and other acoustic measures. Journal of Voice, 28(6), 673–680. 10.1016/j.jvoice.2014.01.013, 24836365

[bib122] Jiménez-Jiménez, F. J., Gamboa, J., Nieto, A., Guerrero, J., Orti-Pareja, M., Molina, J. A., García-Albea, E., & Cobeta, I. (1997). Acoustic voice analysis in untreated patients with Parkinson’s disease. Parkinsonism & Related Disorders, 3(2), 111–116. 10.1016/S1353-8020(97)00007-2, 18591065

[bib123] Kaipa, R., McLain, N., & Kaipa, R. M. (2024). Altered auditory feedback improves speech intelligibility in individuals diagnosed with Parkinson’s disease: A pilot study. Folia Phoniatrica et Logopaedica, 76(5), 420–430. 10.1159/000535559, 38035551

[bib124] Kaji, R., Urushihara, R., Murase, N., Shimazu, H., & Goto, S. (2005). Abnormal sensory gating in basal ganglia disorders. Journal of Neurology, 252(Suppl. 4), IV13–IV16. 10.1007/S00415-005-4004-9, 16222432

[bib125] Kanazawa, Y., Kishimoto, Y., Tateya, I., Ishii, T., Sanuki, T., Hiroshiba, S., Aso, T., Omori, K., & Nakamura, K. (2020). Hyperactive sensorimotor cortex during voice perception in spasmodic dysphonia. Scientific Reports, 10(1), 17298. 10.1038/S41598-020-73450-0, 33057071 PMC7566443

[bib126] Kapsner-Smith, M. R., Abur, D., Eadie, T. L., & Stepp, C. E. (2024). Test-retest reliability of behavioral assays of feedforward and feedback auditory-motor control of voice and articulation. Journal of Speech, Language, and Hearing Research, 67(1), 34–48. 10.1044/2023_JSLHR-23-00038, 37992404 PMC11000789

[bib127] Karlin, R., & Parrell, B. (2026). Speakers with cerebellar ataxia do not adapt speech segment durations in response to durationally altered auditory feedback. Journal of Neuroscience, 46(29), e2289252026. 10.1523/JNEUROSCI.2289-25.2026, 42362378 PMC13393544

[bib128] Karlsson, F., & Hartelius, L. (2019). How well does diadochokinetic task performance predict articulatory imprecision? Differentiating individuals with Parkinson’s disease from control subjects. Folia Phoniatrica et Logopaedica, 71(5–6), 251–260. 10.1159/000498851, 31117108

[bib129] Kawato, M. (1999). Internal models for motor control and trajectory planning. Current Opinion in Neurobiology, 9(6), 718–727. 10.1016/S0959-4388(99)00028-8, 10607637

[bib130] Kearney, E., Giles, R., Haworth, B., Faloutsos, P., Baljko, M., & Yunusova, Y. (2017). Sentence-level movements in Parkinson’s disease: Loud, clear, and slow speech. Journal of Speech, Language, and Hearing Research, 60(12), 3426–3440. 10.1044/2017_JSLHR-S-17-0075, 29209727

[bib131] Kearney, E., Nieto-Castañón, A., Weerathunge, H. R., Falsini, R., Daliri, A., Abur, D., Ballard, K. J., Chang, S.-E., Chao, S.-C., Heller Murray, E. S., Scott, T. L., & Guenther, F. H. (2020). A simple 3-parameter model for examining adaptation in speech and voice production. Frontiers in Psychology, 10, 2995. 10.3389/fpsyg.2019.02995, 32038381 PMC6985569

[bib132] Kent, R. D., Duffy, J. R., Slama, A., Kent, J. F., & Clift, A. (2001). Clinicoanatomic studies in dysarthria: Review, critique, and directions for research. Journal of Speech, Language, and Hearing Research, 44(3), 535–551. 10.1044/1092-4388(2001/042), 11407559

[bib133] Kent, R. D., Kent, J. F., Duffy, J. R., Thomas, J. E., Weismer, G., & Stuntebeck, S. (2000). Ataxic dysarthria. Journal of Speech, Language, and Hearing Research, 43(5), 1275–1289. 10.1044/jslhr.4305.1275, 11063247

[bib134] Kent, R. D., Kent, J. F., Rosenbek, J. C., Vorperian, H. K., & Weismer, G. (1997). A speaking task analysis of the dysarthria in cerebellar disease. Folia Phoniatrica et Logopaedica, 49(2), 63–82. 10.1159/000266440, 9197089

[bib135] Khosravani, S., Mahnan, A., Yeh, I.-L., Aman, J. E., Watson, P. J., Zhang, Y., Goding, G., & Konczak, J. (2019). Laryngeal vibration as a non-invasive neuromodulation therapy for spasmodic dysphonia. Scientific Reports, 9(1), 17955. 10.1038/S41598-019-54396-4, 31784618 PMC6884515

[bib136] Kim, Y., Weismer, G., Kent, R. D., & Duffy, J. R. (2009). Statistical models of F2 slope in relation to severity of dysarthria. Folia Phoniatrica et Logopaedica, 61(6), 329–335. 10.1159/000252849, 19864914 PMC2855272

[bib137] Kiran, S., & Larson, C. R. (2001). Effect of duration of pitch-shifted feedback on vocal responses in patients with Parkinson’s disease. Journal of Speech, Language, and Hearing Research, 44(5), 975–987. 10.1044/1092-4388(2001/076), 11708537

[bib138] Kiyuna, A., Maeda, H., Higa, A., Shingaki, K., Uehara, T., & Suzuki, M. (2014). Brain activity related to phonation in young patients with adductor spasmodic dysphonia. Auris, Nasus, Larynx, 41(3), 278–284. 10.1016/j.anl.2013.10.017, 24359705

[bib139] Knowles, T., Adams, S., Abeyesekera, A., Mancinelli, C., Gilmore, G., & Jog, M. (2018). Deep brain stimulation of the subthalamic nucleus parameter optimization for vowel acoustics and speech intelligibility in Parkinson’s disease. Journal of Speech, Language, and Hearing Research, 61(3), 510–524. 10.1044/2017_JSLHR-S-17-0157, 29471373

[bib140] Koda, J., & Ludlow, C. L. (1992). An evaluation of laryngeal muscle activation in patients with voice tremor. Otolaryngology–Head and Neck Surgery, 107(5), 684–696. 10.1177/019459989210700510, 1437206

[bib141] Kostic, V. S., Agosta, F., Sarro, L., Tomić, A., Kresojević, N., Galantucci, S., Svetel, M., Valsasina, P., & Filippi, M. (2016). Brain structural changes in spasmodic dysphonia: A multimodal magnetic resonance imaging study. Parkinsonism & Related Disorders, 25, 78–84. 10.1016/j.parkreldis.2016.02.003, 26876036

[bib142] Kothare, H., Schneider, S., Mizuiri, D., Hinkley, L., Bhutada, A., Ranasinghe, K., Honma, S., Garrett, C., Klein, D., Naunheim, M., Yung, K., Cheung, S., Rosen, C., Courey, M., Nagarajan, S., & Houde, J. (2022). Temporal specificity of abnormal neural oscillations during phonatory events in laryngeal dystonia. Brain Communications, 4(2), fcac031. 10.1093/braincomms/fcac031, 35356032 PMC8962453

[bib143] Krakauer, J. W., Hadjiosif, A. M., Xu, J., Wong, A. L., & Haith, A. M. (2019). Motor learning. Comprehensive Physiology, 9(2), 613–663. 10.1002/j.2040-4603.2019.tb00069.x, 30873583

[bib144] Krüger, M. T., Hu, A., & Honey, C. R. (2020). Deep brain stimulation for spasmodic dysphonia: A blinded comparison of unilateral and bilateral stimulation in two patients. Stereotactic and Functional Neurosurgery, 98(3), 200–205. 10.1159/000507058, 32316007

[bib145] Kundu, B., Schrock, L., Davis, T., & House, P. A. (2018). Thalamic deep brain stimulation for essential tremor also reduces voice tremor. Neuromodulation, 21(8), 748–754. 10.1111/ner.12739, 29232019

[bib146] Kuruvilla-Dugdale, M., Salazar, M., Zhang, A., & Mefferd, A. S. (2020). Detection of articulatory deficits in Parkinson’s disease: Can systematic manipulations of phonetic complexity help? Journal of Speech, Language, and Hearing Research, 63(7), 2084–2098. 10.1044/2020_JSLHR-19-00245, 32598198 PMC7838836

[bib147] Larson, C. R., Altman, K. W., Liu, H., & Hain, T. C. (2008). Interactions between auditory and somatosensory feedback for voice F0 control. Experimental Brain Research, 187(4), 613–621. 10.1007/s00221-008-1330-z, 18340440 PMC2763543

[bib148] Leanderson, R., Meyerson, B. A., & Persson, A. (1972). Lip muscle function in Parkinsonian dysarthria. Acta Oto-Laryngologica, 74(5), 350–357. 10.3109/00016487209128462, 4565338

[bib149] Lechien, J. R., Blecic, S., Huet, K., Delvaux, V., Piccaluga, M., Roland, V., Harmegnies, B., & Saussez, S. (2018). Voice quality outcomes of idiopathic Parkinson’s disease medical treatment: A systematic review. Clinical Otolaryngology, 43(3), 882–903. 10.1111/coa.13082, 29443454

[bib150] Lee, M. S., Lee, M. J., Conte, A., & Berardelli, A. (2018). Abnormal somatosensory temporal discrimination in Parkinson’s disease: Pathophysiological correlates and role in motor control deficits. Clinical Neurophysiology, 129(2), 442–447. 10.1016/j.clinph.2017.11.022, 29304419

[bib151] Leydon, C., Bauer, J. J., & Larson, C. R. (2003). The role of auditory feedback in sustaining vocal vibrato. The Journal of the Acoustical Society of America, 114(3), 1575–1581. 10.1121/1.1603230, 14514211 PMC1769352

[bib152] Li, W., Zhuang, J., Guo, Z., Jones, J. A., Xu, Z., & Liu, H. (2019). Cerebellar contribution to auditory feedback control of speech production: Evidence from patients with spinocerebellar ataxia. Human Brain Mapping, 40(16), 4748–4758. 10.1002/hbm.24734, 31365181 PMC6865477

[bib153] Li, Y., Tan, M., Fan, H., Wang, E. Q., Chen, L., Li, J., Chen, X., & Liu, H. (2021). Neurobehavioral effects of LSVT® LOUD on auditory-vocal integration in Parkinson’s disease: A preliminary study. Frontiers in Neuroscience, 15, 624801. 10.3389/fnins.2021.624801, 33716652 PMC7952622

[bib154] Lin, E., Jiang, J., Hone, S., & Hanson, D. G. (1999). Photoglottographic measures in Parkinson’s disease. Journal of Voice, 13(1), 25–35. 10.1016/S0892-1997(99)80058-7, 10223672

[bib155] Lin, Q., Chang, Y., Liu, P., Jones, J. A., Chen, X., Peng, D., Chen, M., Wu, C., & Liu, H. (2022). Cerebellar continuous theta burst stimulation facilitates auditory-vocal integration in spinocerebellar ataxia. Cerebral Cortex, 32(3), 455–466. 10.1093/cercor/bhab222, 34240142

[bib156] Liu, H., Wang, E. Q., Metman, L. V., & Larson, C. R. (2012). Vocal responses to perturbations in voice auditory feedback in individuals with Parkinson’s disease. PLOS ONE, 7(3), e33629. 10.1371/journal.pone.0033629, 22448258 PMC3308986

[bib157] Logemann, J. A., & Fisher, H. B. (1981). Vocal tract control in Parkinson’s disease: Phonetic feature analysis of misarticulations. Journal of Speech and Hearing Disorders, 46(4), 348–352. 10.1044/jshd.4604.348, 7300262

[bib158] Logemann, J. A., Fisher, H. B., Boshes, B., & Blonsky, E. R. (1978). Frequency and cooccurrence of vocal tract dysfunctions in the speech of a large sample of Parkinson patients. Journal of Speech and Hearing Disorders, 43(1), 47–57. 10.1044/jshd.4301.47, 633872

[bib159] Louis, E. D. (2018). Essential tremor and the cerebellum. Handbook of Clinical Neurology, 155, 245–258. 10.1016/B978-0-444-64189-2.00016-0, 29891062

[bib160] Louis, E. D., & Faust, P. L. (2020a). Essential tremor: The most common form of cerebellar degeneration? Cerebellum & Ataxias, 7, 12. 10.1186/S40673-020-00121-1, 32922824 PMC7427947

[bib161] Louis, E. D., & Faust, P. L. (2020b). Essential tremor within the broader context of other forms of cerebellar degeneration. Cerebellum, 19(6), 879–896. 10.1007/S12311-020-01160-4, 32666285 PMC7593894

[bib162] Louis, E. D., & McCreary, M. (2021). How common is essential tremor? Update on the worldwide prevalence of essential tremor. Tremor and Other Hyperkinetic Movements, 11, 28. 10.5334/tohm.632, 34277141 PMC8269764

[bib163] Lowit, A., Dobinson, C., Timmins, C., Howell, P., & Kröger, B. (2010). The effectiveness of traditional methods and altered auditory feedback in improving speech rate and intelligibility in speakers with Parkinson’s disease. International Journal of Speech-Language Pathology, 12(5), 426–436. 10.3109/17549507.2010.497559, 20626310

[bib164] Ludlow, C. L., Yamashita, T., Schulz, G. M., & Deleyiannis, F. W.-B. (1995). Abnormalities in long latency responses to superior laryngeal nerve stimulation in adductor spasmodic dysphonia. Annals of Otology, Rhinology, and Laryngology, 104(12), 928–935. 10.1177/000348949510401203, 7492063

[bib165] Lukhanina, E., Berezetskaya, N., & Karaban, I. (2010). Paired-pulse inhibition in the auditory cortex in Parkinson’s disease and its dependence on clinical characteristics of the patients. Parkinson’s Disease, 2011, 342151. 10.4061/2011/342151, 21052541 PMC2968419

[bib166] Lukhanina, E. P., Kapustina, M. T., Berezetskaya, N. M., & Karaban, I. N. (2009). Reduction of the postexcitatory cortical inhibition upon paired-click auditory stimulation in patients with Parkinson’s disease. Clinical Neurophysiology, 120(10), 1852–1858. 10.1016/j.clinph.2009.07.040, 19767236

[bib167] Luschei, E. S., Ramig, L. O., Baker, K. L., & Smith, M. E. (1999). Discharge characteristics of laryngeal single motor units during phonation in young and older adults, and in persons with Parkinson disease. Journal of Neurophysiology, 81(5), 2131–2139. 10.1152/jn.1999.81.5.2131, 10322054

[bib168] Ma, J. K.-Y., Schneider, C. B., Hoffmann, R., & Storch, A. (2015). Speech prosody across stimulus types for individuals with Parkinson’s disease. Journal of Parkinson’s Disease, 5(2), 291–299. 10.3233/JPD-140451, 25697957

[bib169] MacDonald, E. N., Goldberg, R., & Munhall, K. G. (2010). Compensations in response to real-time formant perturbations of different magnitudes. The Journal of the Acoustical Society of America, 127(2), 1059–1068. 10.1121/1.3278606, 20136227 PMC2830267

[bib170] Manto, M. (2018). Cerebellar motor syndrome from children to the elderly. Handbook of Clinical Neurology, 154, 151–166. 10.1016/B978-0-444-63956-1.00009-6, 29903437

[bib171] Martel Sauvageau, V., Roy, J.-P., Langlois, M., & Macoir, J. (2015). Impact of the LSVT on vowel articulation and coarticulation in Parkinson’s disease. Clinical Linguistics & Phonetics, 29(6), 424–440. 10.3109/02699206.2015.1012301, 25688915

[bib172] Mefferd, A. S., & Dietrich, M. S. (2019). Tongue- and jaw-specific articulatory underpinnings of reduced and enhanced acoustic vowel contrast in talkers with Parkinson’s disease. Journal of Speech, Language, and Hearing Research, 62(7), 2118–2132. 10.1044/2019_JSLHR-S-MSC18-18-0192, 31306611 PMC6808361

[bib173] Merson, R. M., & Ginsberg, A. P. (1979). Spasmodic dysphonia: Abductor type. A clinical report of acoustic, aerodynamic and perceptual characteristics. Laryngoscope, 89(1), 129–139. 10.1288/00005537-197901000-00014, 423646

[bib174] Miall, R. C., & Wolpert, D. M. (1996). Forward models for physiological motor control. Neural Networks, 9(8), 1265–1279. 10.1016/S0893-6080(96)00035-4, 12662535

[bib175] Midi, I., Dogan, M., Koseoglu, M., Can, G., Sehitoglu, M. A., & Gunal, D. I. (2008). Voice abnormalities and their relation with motor dysfunction in Parkinson’s disease. Acta Neurologica Scandinavica, 117(1), 26–34. 10.1111/J.1600-0404.2007.00965.X, 18031561

[bib176] Milosevic, L., Kalia, S. K., Hodaie, M., Lozano, A. M., Popovic, M. R., & Hutchison, W. D. (2018). Physiological mechanisms of thalamic ventral intermediate nucleus stimulation for tremor suppression. Brain, 141(7), 2142–2155. 10.1093/brain/awy139, 29878147 PMC6022553

[bib177] Mollaei, F., Shiller, D. M., Baum, S. R., & Gracco, V. L. (2016). Sensorimotor control of vocal pitch and formant frequencies in Parkinson’s disease. Brain Research, 1646, 269–277. 10.1016/j.brainres.2016.06.013, 27288701 PMC4975987

[bib178] Mollaei, F., Shiller, D. M., Baum, S. R., & Gracco, V. L. (2019). The relationship between speech perceptual discrimination and speech production in Parkinson’s disease. Journal of Speech, Language, and Hearing Research, 62(12), 4256–4268. 10.1044/2019_JSLHR-S-18-0425, 31738857

[bib179] Mollaei, F., Shiller, D. M., Baum, S. R., & Gracco, V. L. (2022). Early auditory responses to speech sounds in Parkinson’s disease: Preliminary data. Scientific Reports, 12(1), 1019. 10.1038/S41598-022-05128-8, 35046514 PMC8770631

[bib180] Mollaei, F., Shiller, D. M., & Gracco, V. L. (2013). Sensorimotor adaptation of speech in Parkinson’s disease. Movement Disorders, 28(12), 1668–1674. 10.1002/mds.25588, 23861349 PMC3812368

[bib181] Mongeon, D., Blanchet, P., & Messier, J. (2013). Impact of Parkinson’s disease and dopaminergic medication on adaptation to explicit and implicit visuomotor perturbations. Brain and Cognition, 81(2), 271–282. 10.1016/j.bandc.2012.12.001, 23313834

[bib182] Mücke, D., Becker, J., Barbe, M. T., Meister, I., Liebhart, L., Roettger, T. B., Dembek, T., Timmermann, L., & Grice, M. (2014). The effect of deep brain stimulation on the speech motor system. Journal of Speech, Language, and Hearing Research, 57(4), 1206–1218. 10.1044/2014_JSLHR-S-13-0155, 24686442

[bib183] Mücke, D., Hermes, A., Roettger, T. B., Becker, J., Niemann, H., Dembek, T. A., Timmermann, L., Visser-Vandewalle, V., Fink, G. R., Grice, M., & Barbe, M. T. (2018). The effects of thalamic deep brain stimulation on speech dynamics in patients with essential tremor: An articulographic study. PLoS ONE, 13(1), e0191359. 10.1371/journal.pone.0191359, 29360867 PMC5779681

[bib184] Nakayama, K., Yamamoto, T., Oda, C., Sato, M., Murakami, T., & Horiguchi, S. (2020). Effectiveness of Lee Silverman Voice Treatment® LOUD on Japanese-speaking patients with Parkinson’s disease. Rehabilitation Research and Practice, 2020, 6585264. 10.1155/2020/6585264, 32411475 PMC7204176

[bib185] Narayana, S., Fox, P. T., Zhang, W., Franklin, C., Robin, D. A., Vogel, D., & Ramig, L. O. (2010). Neural correlates of efficacy of voice therapy in Parkinson’s disease identified by performance–correlation analysis. Human Brain Mapping, 31(2), 222–236. 10.1002/hbm.20859, 19639554 PMC2811230

[bib186] Nelson, A. J., Hoque, T., Gunraj, C., & Chen, R. (2018). Altered somatosensory processing in Parkinson’s disease and modulation by dopaminergic medications. Parkinsonism & Related Disorders, 53, 76–81. 10.1016/j.parkreldis.2018.05.002, 29764720

[bib187] Nelson, A. J., Premji, A., Rai, N., Hoque, T., Tommerdahl, M., & Chen, R. (2012). Dopamine alters tactile perception in Parkinson’s disease. Canadian Journal of Neurological Sciences, 39(1), 52–57. 10.1017/S0317167100012683, 22384496

[bib188] Neumann, W.-J., Horn, A., Ewert, S., Huebl, J., Brücke, C., Slentz, C., Schneider, G.-H., & Kühn, A. A. (2017). A localized pallidal physiomarker in cervical dystonia. Annals of Neurology, 82(6), 912–924. 10.1002/ana.25095, 29130551

[bib189] Niziolek, C. A., Nagarajan, S. S., & Houde, J. F. (2013). What does motor efference copy represent? Evidence from speech production. Journal of Neuroscience, 33(41), 16110–16116. 10.1523/JNEUROSCI.2137-13.2013, 24107944 PMC3792453

[bib190] Noffs, G., Boonstra, F. M. C., Perera, T., Kolbe, S. C., Stankovich, J., Butzkueven, H., Evans, A., Vogel, A. P., & van der Walt, A. (2020). Acoustic speech analytics are predictive of cerebellar dysfunction in multiple sclerosis. Cerebellum, 19(5), 691–700. 10.1007/S12311-020-01151-5, 32556973

[bib191] Nolano, M., Provitera, V., Crisci, C., Saltalamacchia, A. M., Wendelschafer-Crabb, G., Kennedy, W. R., Filla, A., Santoro, L., & Caruso, G. (2001). Small fibers involvement in Friedreich’s ataxia. Annals of Neurology, 50(1), 17–25. 10.1002/ana.1283, 11456305

[bib192] Pan, M.-K., & Kuo, S.-H. (2022). Essential tremor: Clinical perspectives and pathophysiology. Journal of the Neurological Sciences, 435, 120198. 10.1016/j.jns.2022.120198, 35299120 PMC10363990

[bib193] Papavassiliou, E., Rau, G., Heath, S., Abosch, A., Barbaro, N. M., Larson, P. S., Lamborn, K., & Starr, P. A. (2004). Thalamic deep brain stimulation for essential tremor: Relation of lead location to outcome. Neurosurgery, 54(5), 1120–1130. 10.1227/01.NEU.0000119329.66931.9E, 15113466

[bib194] Parrell, B., Agnew, Z., Nagarajan, S., Houde, J., & Ivry, R. B. (2017). Impaired feedforward control and enhanced feedback control of speech in patients with cerebellar degeneration. Journal of Neuroscience, 37(38), 9249–9258. 10.1523/JNEUROSCI.3363-16.2017, 28842410 PMC5607467

[bib195] Parrell, B., & Houde, J. (2019). Modeling the role of sensory feedback in speech motor control and learning. Journal of Speech, Language, and Hearing Research, 62(8S), 2963–2985). 10.1044/2019_JSLHR-S-CSMC7-18-0127, 31465712 PMC6813034

[bib196] Parrell, B., Kim, H. E., Breska, A., Saxena, A., & Ivry, R. (2021). Differential effects of cerebellar degeneration on feedforward versus feedback control across speech and reaching movements. Journal of Neuroscience, 41(42), 8779–8789. 10.1523/JNEUROSCI.0739-21.2021, 34446570 PMC8528499

[bib197] Parrell, B., Ramanarayanan, V., Nagarajan, S., & Houde, J. (2019). The FACTS model of speech motor control: Fusing state estimation and task-based control. PLoS Computational Biology, 15(9), e1007321. 10.1371/journal.pcbi.1007321, 31479444 PMC6743785

[bib198] Patel, R. R., Zauber, S. E., Yadav, A. P., Witt, T. C., Halum, S., & Gupta, K. (2022). Globus pallidus interna and ventral intermediate nucleus of the thalamus deep brain stimulation for adductor laryngeal dystonia: A case report of blinded analyses of objective voice outcomes in 2 patients. Neurosurgery, 90(4), 457–463. 10.1227/NEU.0000000000001851, 35138294

[bib199] Perestelo-Pérez, L., Rivero-Santana, A., Pérez-Ramos, J., Serrano-Pérez, P., Panetta, J., & Hilarion, P. (2014). Deep brain stimulation in Parkinson’s disease: Meta-analysis of randomized controlled trials. Journal of Neurology, 261(11), 2051–2060. 10.1007/S00415-014-7254-6, 24487826

[bib200] Perez, K. S., Ramig, L. O., Smith, M. E., & Dromey, C. (1996). The Parkinson larynx: Tremor and videostroboscopic findings. Journal of Voice, 10(4), 354–361. 10.1016/S0892-1997(96)80027-0, 8943139

[bib201] Petry-Schmelzer, J. N., Jergas, H., Thies, T., Steffen, J. K., Reker, P., Dafsari, H. S., Mücke, D., Fink, G. R., Visser-Vandewalle, V., Dembek, T. A., & Barbe, M. T. (2021). Network fingerprint of stimulation-induced speech impairment in essential tremor. Annals of Neurology, 89(2), 315–326. 10.1002/ana.25958, 33201528

[bib202] Phokaewvarangkul, O., Boonpang, K., & Bhidayasiri, R. (2019). Subthalamic deep brain stimulation aggravates speech problems in Parkinson’s disease: Objective and subjective analysis of the influence of stimulation frequency and electrode contact location. Parkinsonism & Related Disorders, 66, 110–116. 10.1016/j.parkreldis.2019.07.020, 31327627

[bib203] Pisani, V., Sisto, R., Moleti, A., Di Mauro, R., Pisani, A., Brusa, L., Altavista, M. C., Stanzione, P., & Di Girolamo, S. (2015). An investigation of hearing impairment in de-novo Parkinson’s disease patients: A preliminary study. Parkinsonism & Related Disorders, 21(8), 987–991. 10.1016/j.parkreldis.2015.06.007, 26071125

[bib204] Poologaindran, A., Ivanishvili, Z., Morrison, M. D., Rammage, L. A., Sandhu, M. K., Polyhronopoulos, N. E., & Honey, C. R. (2018). The effect of unilateral thalamic deep brain stimulation on the vocal dysfunction in a patient with spasmodic dysphonia: Interrogating cerebellar and pallidal neural circuits. Journal of Neurosurgery, 128(2), 575–582. 10.3171/2016.10.JNS161025, 28304188

[bib205] Portnoy, R. A., & Aronson, A. E. (1982). Diadochokinetic syllable rate and regularity in normal and in spastic and ataxic dysarthric subjects. Journal of Speech and Hearing Disorders, 47(3), 324–328. 10.1044/jshd.4703.324, 7186569

[bib206] Postuma, R. B., Berg, D., Stern, M., Poewe, W., Olanow, C. W., Oertel, W., Obeso, J., Marek, K., Litvan, I., Lang, A. E., Halliday, G., Goetz, C. G., Gasser, T., Dubois, B., Chan, P., Bloem, B. R., Adler, C. H., & Deuschl, G. (2015). MDS clinical diagnostic criteria for Parkinson’s disease. Movement Disorders, 30(12), 1591–1601. 10.1002/mds.26424, 26474316

[bib207] Pringsheim, T., Day, G. S., Smith, D. B., Rae-Grant, A., Licking, N., Armstrong, M. J., de Bie, R. M. A., Roze, E., Miyasaki, J. M., Hauser, R. A., Espay, A. J., Martello, J. P., Gurwell, J. A., Billinghurst, L., Sullivan, K., Fitts, M. S., Cothros, N., Hall, D. A., Rafferty, M., … Guideline Subcommittee of the AAN. (2021). Dopaminergic therapy for motor symptoms in early Parkinson disease practice guideline summary: A report of the AAN guideline subcommittee. Neurology, 97(20), 942–957. 10.1212/WNL.0000000000012868, 34782410 PMC8672433

[bib208] Purcell, D. W., & Munhall, K. G. (2006). Compensation following real-time manipulation of formants in isolated vowels. The Journal of the Acoustical Society of America, 119(4), 2288–2297. 10.1121/1.2173514, 16642842

[bib209] Putzel, G. G., Battistella, G., Rumbach, A. F., Ozelius, L. J., Sabuncu, M. R., & Simonyan, K. (2018). Polygenic risk of spasmodic dysphonia is associated with vulnerable sensorimotor connectivity. Cerebral Cortex, 28(1), 158–166. 10.1093/cercor/bhw363, 29117296 PMC6059246

[bib210] Quine, D. B., Regan, D., & Murray, T. J. (1984). Degraded discrimination between speech-like sounds by patients with multiple sclerosis and Friedreich’s ataxia. Brain, 107(4), 1113–1122. 10.1093/brain/107.4.1113, 6509311

[bib211] Raharjo, I., Kothare, H., Nagarajan, S. S., & Houde, J. F. (2021). Speech compensation responses and sensorimotor adaptation to formant feedback perturbations. The Journal of the Acoustical Society of America, 149(2), 1147–1161. 10.1121/10.0003440, 33639824 PMC7892200

[bib212] Rahn, D. A., 3rd, Chou, M., Jiang, J. J., & Zhang, Y. (2007). Phonatory impairment in Parkinson’s disease: Evidence from nonlinear dynamic analysis and perturbation analysis. Journal of Voice, 21(1), 64–71. 10.1016/j.jvoice.2005.08.011, 16377130

[bib213] Railo, H., Nokelainen, N., Savolainen, S., & Kaasinen, V. (2020). Deficits in monitoring self-produced speech in Parkinson’s disease. Clinical Neurophysiology, 131(9), 2140–2147. 10.1016/j.clinph.2020.05.038, 32682241

[bib214] Ramig, L. O., Sapir, S., Fox, C., & Countryman, S. (2001). Changes in vocal loudness following intensive voice treatment (LSVT) in individuals with Parkinson’s disease: A comparison with untreated patients and normal age-matched controls. Movement Disorders, 16(1), 79–83. 10.1002/1531-8257(200101)16:1<79::aid-mds1013>3.0.co;2-h, 11215597

[bib215] Ramos, V. F. M. L., Karp, B. I., & Hallett, M. (2014). Tricks in dystonia: Ordering the complexity. Journal of Neurology, Neurosurgery, and Psychiatry, 85(9), 987–993. 10.1136/jnnp-2013-306971, 24487380 PMC4747630

[bib216] Ranasinghe, K. G., Gill, J. S., Kothare, H., Beagle, A. J., Mizuiri, D., Honma, S. M., Gorno-Tempini, M. L., Miller, B. L., Vossel, K. A., Nagarajan, S. S., & Houde, J. F. (2017). Abnormal vocal behavior predicts executive and memory deficits in Alzheimer’s disease. Neurobiology of Aging, 52, 71–80. 10.1016/j.neurobiolaging.2016.12.020, 28131013 PMC5359035

[bib217] Ranasinghe, K. G., Kothare, H., Kort, N., Hinkley, L. B., Beagle, A. J., Mizuiri, D., Honma, S. M., Lee, R., Miller, B. L., Gorno-Tempini, M. L., Vossel, K. A., Houde, J. F., & Nagarajan, S. S. (2019). Neural correlates of abnormal auditory feedback processing during speech production in Alzheimer’s disease. Scientific Reports, 9(1), 5686. 10.1038/s41598-019-41794-x, 30952883 PMC6450891

[bib218] Rance, G., Corben, L., Barker, E., Carew, P., Chisari, D., Rogers, M., Dowell, R., Jamaluddin, S., Bryson, R., & Delatycki, M. B. (2010). Auditory perception in individuals with Friedreich’s ataxia. Audiology & Neuro-Otology, 15(4), 229–240. 10.1159/000255341, 19893304

[bib219] Rance, G., Fava, R., Baldock, H., Chong, A., Barker, E., Corben, L., & Delatycki, M. B. (2008). Speech perception ability in individuals with Friedreich ataxia. Brain, 131(8), 2002–2012. 10.1093/brain/awn104, 18515321

[bib220] Rangwala, R., Subrahmanya, A., Brent, K., Gupta, S., Ranasinghe, K., Dale, C. L., Gaines, J. L., Pongos, A. L., Rosen, C. A., Schneider, S. L., Barkmeier-Kraemer, J., Simonyan, K., Houde, J. F., & Nagarajan, S. S. (2026). Enhanced pitch centering in individuals with laryngeal dystonia. Frontiers in Human Neuroscience, 20, 1688947. 10.3389/fnhum.2026.1688947, 41834902 PMC12979472

[bib221] Richardson, K. C., & Sussman, J. E. (2019). Intensity resolution in individuals with Parkinson’s disease: Sensory and auditory memory limitations. Journal of Speech, Language, and Hearing Research, 62(9), 3564–3581. 10.1044/2019_JSLHR-H-18-0424, 31513750

[bib222] Ridding, M. C., Inzelberg, R., & Rothwell, J. C. (1995). Changes in excitability of motor cortical circuitry in patients with Parkinson’s disease. Annals of Neurology, 37(2), 181–188. 10.1002/ana.410370208, 7847860

[bib223] Rosen, K. M., Kent, R. D., Delaney, A. L., & Duffy, J. R. (2006). Parametric quantitative acoustic analysis of conversation produced by speakers with dysarthria and healthy speakers. Journal of Speech, Language, and Hearing Research, 49(2), 395–411. 10.1044/1092-4388(2006/031), 16671852

[bib224] Rossini, P. M., Filippi, M. M., & Vernieri, F. (1998). Neurophysiology of sensorimotor integration in Parkinson’s disease. Clinical Neuroscience, 5(2), 121–130. 10785838

[bib225] Roy, N., Gouse, M., Mauszycki, S. C., Merrill, R. M., & Smith, M. E. (2005). Task specificity in adductor spasmodic dysphonia versus muscle tension dysphonia. Laryngoscope, 115(2), 311–316. 10.1097/01.MLG.0000154739.48314.EE, 15689757

[bib226] Roy, N., Mazin, A., & Awan, S. N. (2014). Automated acoustic analysis of task dependency in adductor spasmodic dysphonia versus muscle tension dysphonia. Laryngoscope, 124(3), 718–724. 10.1002/lary.24362, 23946147

[bib227] Roy, N., Whitchurch, M., Merrill, R. M., Houtz, D., & Smith, M. E. (2008). Differential diagnosis of adductor spasmodic dysphonia and muscle tension dysphonia using phonatory break analysis. Laryngoscope, 118(12), 2245–2253. 10.1097/MLG.0B013E318184577C, 19029863

[bib228] Ruckart, K. W., Moya-Mendez, M. E., Nagatsuka, M., Barry, J. L., Siddiqui, M. S., & Madden, L. L. (2020). Comprehensive evaluation of voice-specific outcomes in patients with essential tremor before and after deep brain stimulation. Journal of Voice, 36(6), 838–846. 10.1016/j.jvoice.2020.09.013, 33071149

[bib229] Rusz, J., Cmejla, R., Ruzickova, H., & Ruzicka, E. (2011). Quantitative acoustic measurements for characterization of speech and voice disorders in early untreated Parkinson’s disease. The Journal of the Acoustical Society of America, 129(1), 350–367. 10.1121/1.3514381, 21303016

[bib230] Rusz, J., Cmejla, R., Tykalova, T., Ruzickova, H., Klempir, J., Majerova, V., Picmausova, J., Roth, J., & Ruzicka, E. (2013). Imprecise vowel articulation as a potential early marker of Parkinson’s disease: Effect of speaking task. The Journal of the Acoustical Society of America, 134(3), 2171–2181. 10.1121/1.4816541, 23967947

[bib231] Rusz, J., Tykalová, T., Klempíř, J., Čmejla, R., & Růžička, E. (2016). Effects of dopaminergic replacement therapy on motor speech disorders in Parkinson’s disease: Longitudinal follow-up study on previously untreated patients. Journal of Neural Transmission, 123(4), 379–387. 10.1007/S00702-016-1515-8, 26843071

[bib232] Rusz, J., Tykalova, T., Ramig, L. O., & Tripoliti, E. (2021). Guidelines for speech recording and acoustic analyses in dysarthrias of movement disorders. Movement Disorders, 36(4), 803–814. 10.1002/mds.28465, 33373483

[bib233] Sabatini, U., Boulanouar, K., Fabre, N., Martin, F., Carel, C., Colonnese, C., Bozzao, L., Berry, I., Montastruc, J. L., Chollet, F., & Rascol, O. (2000). Cortical motor reorganization in akinetic patients with Parkinson’s disease: A functional MRI study. Brain, 123(2), 394–403. 10.1093/brain/123.2.394, 10648446

[bib234] Sandström, L., Blomstedt, P., & Karlsson, F. (2019). Voice tremor response to deep brain stimulation in relation to electrode location in the posterior subthalamic area. World Neurosurgery: X, 3, 100024. 10.1016/j.wnsx.2019.100024, 31225518 PMC6584456

[bib235] Sandström, L., Karlsson, F., & Blomstedt, P. (2018). Unilateral left deep brain stimulation of the caudal zona incerta is equally effective on voice tremor as bilateral stimulation: Evidence from 7 patients with essential tremor. Stereotactic and Functional Neurosurgery, 96(3), 157–161. 10.1159/000489938, 29975951

[bib236] Sanuki, T. (2023). Spasmodic dysphonia: An overview of clinical features and treatment options. Auris, Nasus, Larynx, 50(1), 17–22. 10.1016/j.anl.2022.05.012, 35697560

[bib237] Sapienza, C. M., Cannito, M. P., Murry, T., Branski, R., & Woodson, G. (2002). Acoustic variations in reading produced by speakers with spasmodic dysphonia pre-botox injection and within early stages of post-botox injection. Journal of Speech, Language, and Hearing Research, 45(5), 830–843. 10.1044/1092-4388(2002/067), 12381042

[bib238] Sapienza, C. M., Murry, T., & Brown, W. S., Jr. (1998). Variations in adductor spasmodic dysphonia: Acoustic evidence. Journal of Voice, 12(2), 214–222. 10.1016/S0892-1997(98)80041-6, 9649077

[bib239] Sapienza, C. M., Walton, S., & Murry, T. (1999). Acoustic variations in adductor spasmodic dysphonia as a function of speech task. Journal of Speech, Language, and Hearing Research, 42(1), 127–140. 10.1044/jslhr.4201.127, 10025549

[bib240] Sapienza, C. M., Walton, S., & Murry, T. (2000). Adductor spasmodic dysphonia and muscular tension dysphonia: Acoustic analysis of sustained phonation and reading. Journal of Voice, 14(4), 502–520. 10.1016/S0892-1997(00)80008-9, 11130109

[bib241] Sapir, S. (2014). Multiple factors are involved in the dysarthria associated with Parkinson’s disease: A review with implications for clinical practice and research. Journal of Speech, Language, and Hearing Research, 57(4), 1330–1343. 10.1044/2014_JSLHR-S-13-0039, 24686571

[bib243] Schaefer, S. D., Finitzo, T., Ross, E., Close, L., Reisch, J., Freeman, F., Cannito, M., & Maravilla, K. (1985). Magnetic resonance imaging findings and correlations in spasmodic dysphonia patients. Annals of Otology, Rhinology, and Laryngology, 94(6), 595–601. 10.1177/000348948509400615, 4073738

[bib242] Schaefer, S. D., Gerling, I. J., Finitzo-Hieber, T., & Freeman, F. J. (1983). Brainstem conduction abnormalities in spasmodic dysphonia. Annals of Otology, Rhinology, and Laryngology, 92(1), 59–64. 10.1177/000348948309200114, 6824281

[bib244] Schalling, E., Hammarberg, B., & Hartelius, L. (2007). Perceptual and acoustic analysis of speech in individuals with spinocerebellar ataxia (SCA). Logopedics, Phoniatrics, Vocology, 32(1), 31–46. 10.1080/14015430600789203, 17454658

[bib245] Scheerer, N. E., Jacobson, D. S., & Jones, J. A. (2016). Sensorimotor learning in children and adults: Exposure to frequency-altered auditory feedback during speech production. Neuroscience, 314, 106–115. 10.1016/j.neuroscience.2015.11.037, 26628403

[bib246] Scheerer, N. E., & Jones, J. A. (2012). The relationship between vocal accuracy and variability to the level of compensation to altered auditory feedback. Neuroscience Letters, 529(2), 128–132. 10.1016/j.neulet.2012.09.012, 22995182

[bib247] Schneider, J. S., Diamond, S. G., & Markham, C. H. (1986). Deficits in orofacial sensorimotor function in Parkinson’s disease. Annals of Neurology, 19(3), 275–282. 10.1002/ana.410190309, 3963772

[bib248] Shadmehr, R., & Mussa-Ivaldi, F. A. (1994). Adaptive representation of dynamics during learning of a motor task. Journal of Neuroscience, 14(5), 3208–3224. 10.1523/JNEUROSCI.14-05-03208.1994, 8182467 PMC6577492

[bib249] Shalash, A. S., Hassan, D. M., Elrassas, H. H., Salama, M. M., Méndez-Hernández, E., Salas-Pacheco, J. M., & Arias-Carrión, O. (2017). Auditory- and vestibular-evoked potentials correlate with motor and non-motor features of Parkinson’s disease. Frontiers in Neurology, 8, 55. 10.3389/fneur.2017.00055, 28289399 PMC5326766

[bib250] Simonyan, K., Barkmeier-Kraemer, J., Blitzer, A., Hallett, M., Houde, J. F., Jacobson Kimberley, T., Ozelius, L. J., Pitman, M. J., Richardson, R. M., Sharma, N., Tanner, K., & The NIH/NIDCD Workshop on Research Priorities in Spasmodic Dysphonia/Laryngeal Dystonia. (2021). Laryngeal dystonia: Multidisciplinary update on terminology, pathophysiology, and research priorities. Neurology, 96(21), 989–1001. 10.1212/WNL.0000000000011922, 33858994 PMC8205448

[bib251] Simonyan, K., & Ludlow, C. L. (2010). Abnormal activation of the primary somatosensory cortex in spasmodic dysphonia: An fMRI study. Cerebral Cortex, 20(11), 2749–2759. 10.1093/cercor/bhq023, 20194686 PMC2951850

[bib252] Simonyan, K., & Ludlow, C. L. (2012). Abnormal structure-function relationship in spasmodic dysphonia. Cerebral Cortex, 22(2), 417–425. 10.1093/cercor/bhr120, 21666131 PMC3256408

[bib253] Skodda, S., Grönheit, W., Mancinelli, N., & Schlegel, U. (2013). Progression of voice and speech impairment in the course of Parkinson’s disease: A longitudinal study. Parkinson’s Disease, 2013, 389195. 10.1155/2013/389195, 24386590 PMC3872441

[bib254] Skodda, S., Grönheit, W., & Schlegel, U. (2011). Intonation and speech rate in Parkinson’s disease: General and dynamic aspects and responsiveness to levodopa admission. Journal of Voice, 25(4), e199–e205. 10.1016/j.jvoice.2010.04.007, 21051196

[bib255] Skodda, S., & Schlegel, U. (2008). Speech rate and rhythm in Parkinson’s disease. Movement Disorders, 23(7), 985–992. 10.1002/mds.21996, 18383114

[bib256] Slis, A., & Parrell, B. (2026). Sensorimotor adaptation of vocal pitch is impaired in cerebellar ataxia. Journal of Cognitive Neuroscience, 38(10), 1985–2007. 10.1162/JOCN.a.2630, 42341286 PMC13569481

[bib257] Statton, M. A., Vazquez, A., Morton, S. M., Vasudevan, E. V. L., & Bastian, A. J. (2018). Making sense of cerebellar contributions to perceptual and motor adaptation. Cerebellum, 17(2), 111–121. 10.1007/S12311-017-0879-0, 28840476 PMC5826770

[bib258] Steeves, T. D., Day, L., Dykeman, J., Jette, N., & Pringsheim, T. (2012). The prevalence of primary dystonia: A systematic review and meta-analysis. Movement Disorders, 27(14), 1789–1796. 10.1002/mds.25244, 23114997

[bib259] Tabari, F., Berger, J. I., Flouty, O., Copeland, B., Greenlee, J. D., & Johari, K. (2024). Speech, voice, and language outcomes following deep brain stimulation: A systematic review. PLOS ONE, 19(5), e0302739. 10.1371/journal.pone.0302739, 38728329 PMC11086900

[bib260] Tanaka, Y., Tsuboi, T., Watanabe, H., Torii, J., Nakatsubo, D., Maesawa, S., Sato, M., Hiraga, K., Satake, Y., Yokoi, K., Hattori, M., Kawabata, K., Hara, K., Yamamoto, M., Sobue, G., & Katsuno, M. (2021). Instability of speech in Parkinson disease patients with subthalamic nucleus deep brain stimulation. Parkinsonism & Related Disorders, 93, 8–11. 10.1016/j.parkreldis.2021.10.029, 34753003

[bib261] Taylor, J. A., Klemfuss, N. M., & Ivry, R. B. (2010). An explicit strategy prevails when the cerebellum fails to compute movement errors. Cerebellum, 9(4), 580–586. 10.1007/S12311-010-0201-X, 20697860 PMC2996538

[bib262] Thies, T., Mücke, D., Dano, R., & Barbe, M. T. (2021). Levodopa-based changes on vocalic speech movements during prosodic prominence marking. Brain Sciences, 11(5), 594. 10.3390/brainsci11050594, 34064356 PMC8147761

[bib263] Thomas, A., Mirza, N., & Eliades, S. J. (2021). Auditory feedback control of vocal pitch in spasmodic dysphonia. Laryngoscope, 131(9), 2070–2075. 10.1002/lary.29254, 33169850

[bib264] Tinazzi, M., Fasano, A., Di Matteo, A., Conte, A., Bove, F., Bovi, T., Peretti, A., Defazio, G., Fiorio, M., & Berardelli, A. (2013). Temporal discrimination in patients with dystonia and tremor and patients with essential tremor. Neurology, 80(1), 76–84. 10.1212/WNL.0B013E31827B1A54, 23243072

[bib265] Tinazzi, M., Fiorio, M., Fiaschi, A., Rothwell, J. C., & Bhatia, K. P. (2009). Sensory functions in dystonia: Insights from behavioral studies. Movement Disorders, 24(10), 1427–1436. 10.1002/mds.22490, 19306289

[bib266] Tjaden, K., Lam, J., & Wilding, G. (2013). Vowel acoustics in Parkinson’s disease and multiple sclerosis: Comparison of clear, loud, and slow speaking conditions. Journal of Speech, Language, and Hearing Research, 56(5), 1485–1502. 10.1044/1092-4388(2013/12-0259), 23838989 PMC5572218

[bib267] Tjaden, K., & Wilding, G. E. (2004). Rate and loudness manipulations in dysarthria: Acoustic and perceptual findings. Journal of Speech, Language, and Hearing Research, 47(4), 766–783. 10.1044/1092-4388(2004/058), 15324285

[bib268] Tourville, J. A., & Guenther, F. H. (2011). The DIVA model: A neural theory of speech acquisition and production. Language and Cognitive Processes, 26(7), 952–981. 10.1080/01690960903498424, 23667281 PMC3650855

[bib269] Tourville, J. A., Reilly, K. J., & Guenther, F. H. (2008). Neural mechanisms underlying auditory feedback control of speech. NeuroImage, 39(3), 1429–1443. 10.1016/j.neuroimage.2007.09.054, 18035557 PMC3658624

[bib270] Troche, J., Troche, M. S., Berkowitz, R., Grossman, M., & Reilly, J. (2012). Tone discrimination as a window into acoustic perceptual deficits in Parkinson’s disease. American Journal of Speech-Language Pathology, 21(3), 258–263. 10.1044/1058-0360(2012/11-0007), 22442285 PMC3592977

[bib271] Tykalova, T., Novotny, M., Ruzicka, E., Dusek, P., & Rusz, J. (2022). Short-term effect of dopaminergic medication on speech in early-stage Parkinson’s disease. NPJ Parkinson’s Disease, 8(1), 22. 10.1038/S41531-022-00286-Y, 35256614 PMC8901688

[bib272] Tykalová, T., Rusz, J., Čmejla, R., Klempíř, J., Růžičková, H., Roth, J., & Růžička, E. (2015). Effect of dopaminergic medication on speech dysfluency in Parkinson’s disease: A longitudinal study. Journal of Neural Transmission, 122(8), 1135–1142. 10.1007/S00702-015-1363-Y, 25583417

[bib273] Tykalova, T., Rusz, J., Klempir, J., Cmejla, R., & Ruzicka, E. (2017). Distinct patterns of imprecise consonant articulation among Parkinson’s disease, progressive supranuclear palsy and multiple system atrophy. Brain and Language, 165, 1–9. 10.1016/j.bandl.2016.11.005, 27894006

[bib274] Valkonen, K., Mäkelä, J. P., Airaksinen, K., Nurminen, J., Kivisaari, R., Renvall, H., & Pekkonen, E. (2022). Deep brain stimulation of subthalamic nucleus modulates cortical auditory processing in advanced Parkinson’s disease. PLOS ONE, 17(2), e0264333. 10.1371/journal.pone.0264333, 35202426 PMC8870490

[bib275] van Brenk, F., Stipancic, K. L., Rohl, A. H., Corcos, D. M., Tjaden, K., & Greenlee, J. D. W. (2024). No differential effects of subthalamic nucleus vs. globus pallidus deep brain stimulation in Parkinson’s disease: Speech acoustic and perceptual findings. IBRO Neuroscience Reports, 16, 361–367. 10.1016/j.ibneur.2024.01.015, 38425546 PMC10902141

[bib276] van Esch, B. F., Wegner, I., Stegeman, I., & Grolman, W. (2017). Effect of botulinum toxin and surgery among spasmodic dysphonia patients. Otolaryngology–Head and Neck Surgery, 156(2), 238–254. 10.1177/0194599816675320, 27803079

[bib277] Vandana, V. P., Darshini, J. K., Vikram, V. H., Nitish, K., Kumar, P. P., & Ravi, Y. (2021). Speech characteristics of patients with Parkinson’s disease—Does dopaminergic medications have a role? Journal of Neurosciences in Rural Practice, 12(4), 673–679. 10.1055/S-0041-1735249, 34737501 PMC8559083

[bib278] Venkatakrishnan, A., Banquet, J. P., Burnod, Y., & Contreras-Vidal, J. L. (2011). Parkinson’s disease differentially affects adaptation to gradual as compared to sudden visuomotor distortions. Human Movement Science, 30(4), 760–769. 10.1016/j.humov.2010.08.020, 21414678 PMC3203839

[bib279] Villacorta, V. M., Perkell, J. S., & Guenther, F. H. (2007). Sensorimotor adaptation to feedback perturbations of vowel acoustics and its relation to perception. The Journal of the Acoustical Society of America, 122(4), 2306–2319. 10.1121/1.2773966, 17902866

[bib280] Vogel, A. P., Wardrop, M. I., Folker, J. E., Synofzik, M., Corben, L. A., Delatycki, M. B., & Awan, S. N. (2017). Voice in Friedreich ataxia. Journal of Voice, 31(2), 243.e9–243.e19. 10.1016/j.jvoice.2016.04.015, 27501923

[bib281] von Cramon, D. (1981). Bilateral cerebellar dysfunctions in a unilateral meso-diencephalic lesion. Journal of Neurology, Neurosurgery, and Psychiatry, 44(4), 361–363. 10.1136/jnnp.44.4.361, 7241166 PMC490964

[bib282] Weerathunge, H. R., Alzamendi, G. A., Cler, G. J., Guenther, F. H., Stepp, C. E., & Zañartu, M. (2022). LaDIVA: A neurocomputational model providing laryngeal motor control for speech acquisition and production. PLoS Computational Biology, 18(6), e1010159. 10.1371/journal.pcbi.1010159, 35737706 PMC9258861

[bib283] Weerathunge, H. R., Tomassi, N. E., & Stepp, C. E. (2022). What can altered auditory feedback paradigms tell us about vocal motor control in individuals with voice disorders? Perspectives of the ASHA Special Interest Groups, 7(3), 959–976. 10.1044/2022_PERSP-21-00195, 37397620 PMC10312128

[bib284] Whitfield, J. A., & Goberman, A. M. (2014). Articulatory-acoustic vowel space: Application to clear speech in individuals with Parkinson’s disease. Journal of Communication Disorders, 51, 19–28. 10.1016/j.jcomdis.2014.06.005, 25074511

[bib285] Whitfield, J. A., & Mehta, D. D. (2019). Examination of clear speech in Parkinson disease using measures of working vowel space. Journal of Speech, Language, and Hearing Research, 62(7), 2082–2098. 10.1044/2019_JSLHR-S-MSC18-18-0189, 31306606

[bib286] Wolfe, V. I., & Bacon, M. (1976). Spectrographic comparison of two types of spastic dysphonia. Journal of Speech and Hearing Disorders, 41(3), 325–332. 10.1044/jshd.4103.325, 950791

[bib287] Wolpert, D. M., Ghahramani, Z., & Jordan, M. I. (1995). An internal model for sensorimotor integration. Science, 269(5232), 1880–1882. 10.1126/science.7569931, 7569931

[bib288] Wu, T., & Hallett, M. (2013). The cerebellum in Parkinson’s disease. Brain, 136(3), 696–709. 10.1093/brain/aws360, 23404337 PMC7273201

[bib289] Xie, T., Bernard, J., & Warnke, P. (2012). Post subthalamic area deep brain stimulation for tremors: A mini-review. Translational Neurodegeneration, 1(1), 20. 10.1186/2047-9158-1-20, 23210767 PMC3534556

[bib290] Xu, M., Tachibana, R. O., Okanoya, K., Hagiwara, H., Hashimoto, R.-I., & Homae, F. (2020). Unconscious and distinctive control of vocal pitch and timbre during altered auditory feedback. Frontiers in Psychology, 11, 1224. 10.3389/fpsyg.2020.01224, 32581975 PMC7294928

[bib291] Yamada, K., Akazawa, K., Yuen, S., Goto, M., Matsushima, S., Takahata, A., Nakagawa, M., Mineura, K., & Nishimura, T. (2010). MR imaging of ventral thalamic nuclei. American Journal of Neuroradiology, 31(4), 732–735. 10.3174/ajnr.A1870, 19926703 PMC7964215

[bib292] Yanagida, S., Nishizawa, N., Mizoguchi, K., Hatakeyama, H., & Fukuda, S. (2015). Voice onset time for the word-initial voiceless consonant /t/ in Japanese spasmodic dysphonia—A comparison with normal controls. Journal of Voice, 29(4), 450–454. 10.1016/j.jvoice.2014.09.028, 25944292

[bib293] Yang, S., Wang, F., Yang, L., Xu, F., Luo, M., Chen, X., Feng, X., & Zou, X. (2020). The physical significance of acoustic parameters and its clinical significance of dysarthria in Parkinson’s disease. Scientific Reports, 10(1), 11776. 10.1038/S41598-020-68754-0, 32678256 PMC7366911

[bib294] Yücetürk, A., Yılmaz, H., Egˇrilmez, M., & Karaca, S. (2002). Voice analysis and videolaryngostroboscopy in patients with Parkinson’s disease. European Archives of Oto-Rhino-Laryngology, 259(6), 290–293. 10.1007/S00405-002-0462-1, 12115074

[bib295] Zarzur, A. P., de Campos Duprat, A., Cataldo, B. O., Ciampi, D., & Fonoff, E. (2014). Laryngeal electromyography as a diagnostic tool for Parkinson’s disease. Laryngoscope, 124(3), 725–729. 10.1002/lary.24379, 24105813

[bib296] Zarzur, A. P., Duprat, A. C., Shinzato, G., & Eckley, C. A. (2007). Laryngeal electromyography in adults with Parkinson’s disease and voice complaints. Laryngoscope, 117(5), 831–834. 10.1097/MLG.0B013E3180333145, 17473678

[bib297] Zeigelboim, B. S., Teive, H. A. G., Santos, R. S., Arruda, W. O., Jurkiewicz, A. L., Mengelberg, H., França, D., & Marques, J. M. (2013). Audiological evaluation in spinocerebellar ataxia. CoDAS, 25(4), 351–357. 10.1590/S2317-17822013005000001, 24413425

[bib298] Ziegler, W., & Wessel, K. (1996). Speech timing in ataxic disorders: Sentence production and rapid repetitive articulation. Neurology, 47(1), 208–214. 10.1212/WNL.47.1.208, 8710079

[bib299] Zwirner, P., & Barnes, G. J. (1992). Vocal tract steadiness: A measure of phonatory and upper airway motor control during phonation in dysarthria. Journal of Speech and Hearing Research, 35(4), 761–768. 10.1044/jshr.3504.761, 1405531

[bib300] Zwirner, P., Murry, T., & Woodson, G. E. (1993). Perceptual-acoustic relationships in spasmodic dysphonia. Journal of Voice, 7(2), 165–171. 10.1016/S0892-1997(05)80347-9, 8353631

